# IbinA and IbinB regulate the Toll pathway-mediated immune response in *Drosophila melanogaster*

**DOI:** 10.1186/s12915-025-02501-7

**Published:** 2026-01-09

**Authors:** Matthew K. Maasdorp, Susanna Valanne, Laura Vesala, Petra Vornanen, Elina Haukkavaara, Tea Tuomela, Aino Malin, Tiina S. Salminen, Dan Hultmark, Mika Rämet

**Affiliations:** 1 Experimental Immunology Research Group, Faculty of Medicine and Health Technology, Tampere University, Tampere, Finland; 2 Mitochondrial Immunometabolism Research Group, Faculty of Medicine and Health Technology, Tampere University, Tampere, Finland; 3 Department of Molecular Biology, Umeå University, Umeå, Sweden

**Keywords:** *Drosophila melanogaster*, IbinA, IbinB, Infection, Innate immunity, Melanization, Mibin, Toll pathway

## Abstract

**Background:**

To combat infection, an immune system needs to be promptly activated but tightly controlled to avoid destruction of host tissues. IbinA and IbinB are related short peptides with robust expression upon microbial challenge in *Drosophila melanogaster*.

**Results:**

*Ibin* genes are ubiquitously present in flies of the *Drosophila* subgenus *Sophophora*, replacing the likely evolutionarily older, related gene, *Mibin*, which is found across a much wider range of cyclorrhaphan flies and is also upregulated following infection. We observed no direct bactericidal or bacteriostatic activity for IbinA or IbinB in vitro. Using single and double *Ibin* mutant *Drosophila* lines, we examined their roles in development and during microbial infections. *IbinA* is expressed in early pupae, and a lack of *IbinA* and *IbinB* leads to temperature-dependent formation of melanized tissue during metamorphosis, frequently around the trachea. IbinA and IbinB have distinct effects on susceptibility to microbial infection. For example, flies lacking *IbinB* had improved survival when challenged with *Listeria monocytogenes*, an intracellular pathogen, whereas a lack of *IbinA* alone had no effect. RNA sequencing following *L. monocytogenes* infection showed enhanced Toll target gene expression in flies lacking *IbinB*, suggesting that IbinB acts as a negative regulator of the Toll pathway. In contrast, *IbinA* mutants had decreased Toll target gene expression. Correspondingly, *IbinB* mutant flies had improved, and *Ibin*A compromised survival in septic fungal infection, where the Toll pathway has a major role.

**Conclusions:**

Our study provides insight into the roles of IbinA and IbinB in regulation of the immune response in *Drosophila*.

**Supplementary Information:**

The online version contains supplementary material available at 10.1186/s12915-025-02501-7.

## Background

*Drosophila melanogaster* has an elegant innate immune system that includes both cellular and humoral arms [1]. The cellular immune response involves mechanisms such as pathogen recognition, phagocytosis of bacteria, encapsulation, and the killing of parasitoid wasps [2–5] and is mediated by *Drosophila* immune cells called hemocytes. The *Drosophila* humoral immune response is initiated by the recognition of pattern-associated molecular patterns (PAMPs) by pattern recognition receptors (PRRs), resulting in the release and nuclear localization of NF-κB transcription factors (Dif, dorsal, and Relish) via the Toll and immune deficiency (Imd) signaling pathways, which are conserved between flies and humans [6–10]. Whereas the microbial recognition mechanisms and the core signaling pathways leading to activation of the humoral immune response via the Toll and Imd pathways have been well established, the immune effector mechanisms are largely yet to be characterized at the molecular level. Some of the induced genes encode secreted peptides with direct antimicrobial properties, like cecropins, attacins, diptericins, insect defensins, drosomycin (Drs), and metchnikowin (Mtk) [11–16], whereas some, like *pirk*, are involved in the regulation of the immune response [17–20]. The role of many infection-inducible genes is, however, currently unknown.

In our previous work, we identified novel immune-induced molecules, namely Induced By INfection (IBIN, CG44404 [CR44404]) and CG45045 (CR45045), which are rapidly induced in immune challenge via both the Toll and the Imd pathways [21]. It was originally thought that IBIN and CG45045 are noncoding RNA molecules, but they have been re-annotated as peptide-encoding genes with strong sequence similarity to each other [21, 22]. Because of their similarity, we have renamed the peptides as IbinA (IBIN, CG44404) and IbinB (CG45045). The *IbinA* gene is predicted to encode a 41 amino acid-long peptide, whereas the *IbinB* gene gives rise to a putative 42 amino acid-long peptide homologous to IbinA. *IbinA* and *IbinB* are highly upregulated by both gram-positive and gram-negative bacteria, as well as when fly larvae are parasitized by *Leptopilina boulardi* wasps [21]. Upon infection, *IbinA* is expressed in the fat body (the functional equivalent of the mammalian liver and adipose tissue and an important immune tissue in the fly), in hemocytes (fly blood cells), and in the gut. Interestingly, ectopic *IbinA* expression in the fat body and in the gut alters expression of sugar metabolism genes and downregulates protein metabolism genes [21]. In addition, others have shown that *IbinA* is induced in female flies upon sight of parasitoid wasps [23], and in male flies upon social isolation [24], suggesting a role for Ibin peptides also in other stress-related situations besides infection. Here, we set out to investigate the roles of IbinA and IbinB in *Drosophila* immunity using deletion mutant lines for the *IbinA* and *IbinB* genes, as well as a double mutant line lacking both genes. We show that *IbinA* and *IbinB* are members of a family of immune-regulated genes found across a wide range of cyclorrhaphan flies and likely originate from a more ancestral immune-inducible gene. In *D. melanogaster*, IbinA and IbinB regulate the humoral immune response. This work adds to the range of previously described negative regulators of the *Drosophila* NF-κB immune pathways, including Pirk [19], Trapid [25], Zfh1 [26], and secreted forms of PGRP [27] for the Imd pathway, and negative Toll pathway regulators including cactus [28], necrotic [29], retromer [30], and the Osa-containing Brahma complex [31].

## Results

### IbinA and IbinB are part of a family of genes conserved across fly species and are induced by infection

**Fig. 1:**
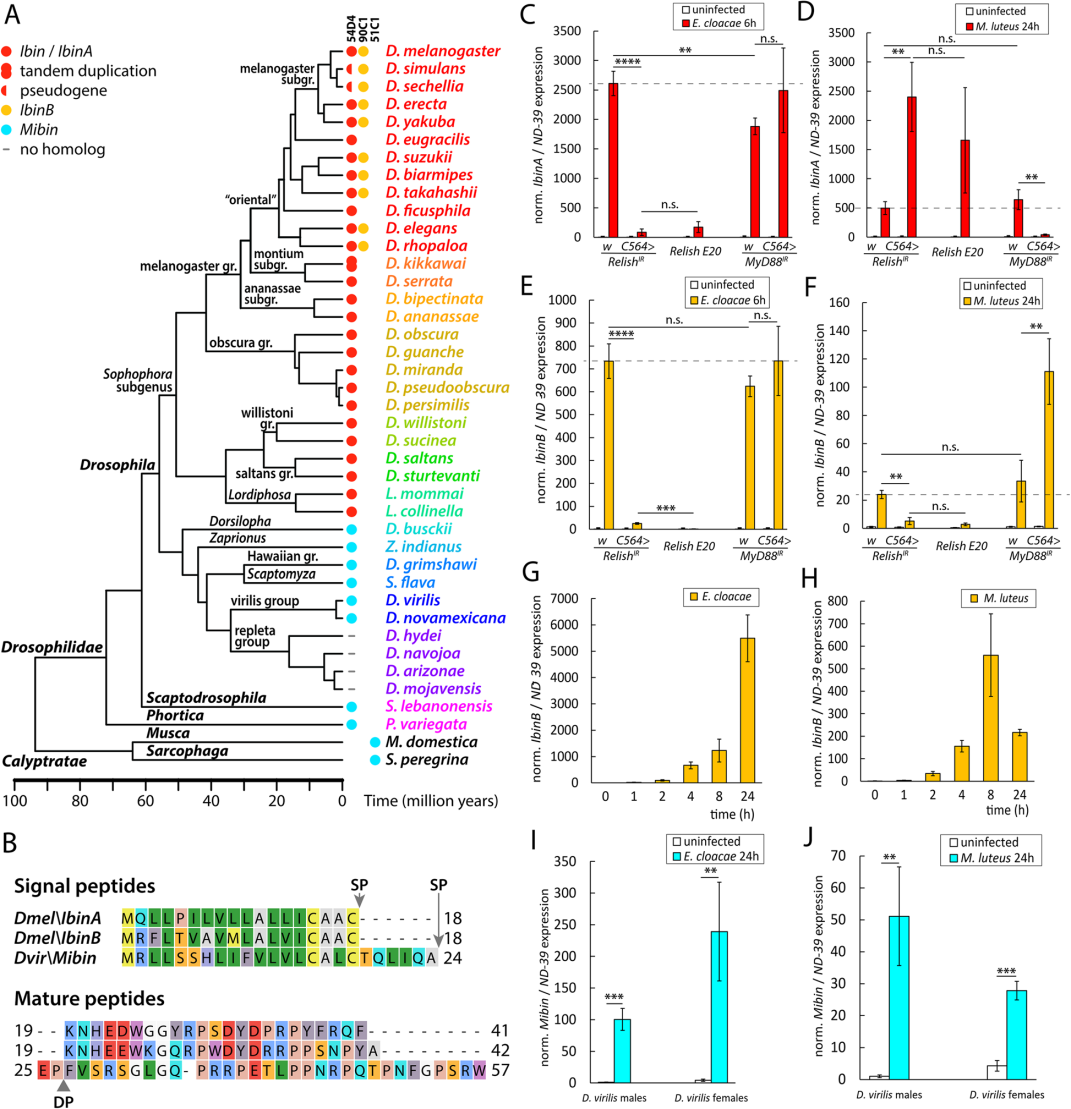
*IbinA* and *IbinB* belong to a family of genes conserved across fly species. **A** Consensus phylogenetic tree showing the occurrence and chromosomal locations (54D4, 90C1 or 51C1) of *IbinA, IbinB*, and *Mibin* orthologs in representative drosophilid flies. Time calibration is based on [33], and the calyptrate outgroup is not time calibrated. **B** Predicted IbinA and IbinB peptide sequences with the predicted cleavage sites for signal peptide (SP, likelihood: 0.998) marked by arrows and for dipeptidyl peptidase (DP) marked by a triangle. *IbinA* or *IbinB* expression in *D. melanogaster* when challenged **C** and **E** with *E. cloacae* or **D** and **F** with *M. luteus*. **G**, **H** Expression patterns of *IbinB* measured within the first 24 h after *D. melanogaster* exposure to *E. cloacae* or *M. luteus*. **I**, **J** *Mibin* expression in *Drosophila virilis* males and females when exposed to *E. cloacae* or *M. luteus*. **C**, **D**, **E**, **F**, **I**, **J** Statistically significant differences are marked with asterisks: *p < 0.05, **p < 0.01, ***p < 0.001, ****p < 0.0001. For fold induction values, expression values in uninfected flies were set to 1

We identified *Ibin* homologs by blastp and tblastn searches in various *Drosophila* species (examples are given in Fig. 1A and Additional file 1: Fig. S1) but only within the subgenus *Sophophora* (including *Lordiphosa* and the willistoni and saltans groups). To get insight into the origin of this apparent novelty, we looked at the corresponding chromosomal regions in other fly species. In *D. melanogaster*, the *IbinA* gene is located between the *CG30109* and *p32* genes on chromosome 2. In terms of the wider chromosomal context, *Ibin* genes are located near the diptericin/attacin loci (Additional file 1: Fig. S2). In species that lack *Ibin* homologs, the corresponding chromosomal locus encodes a proline-rich peptide, which we call “Mother of Ibin” (Mibin, Additional file 1: Fig. S2).

Conserved *Mibin* genes could be identified among most Drosophilidae as well as in other acalyptrate and calyptrate flies, like the housefly (*Musca domestica*) and the flesh fly (*Sarcophaga peregrina*), and even in aschizan flies, like the hoverflies, family Syrphidae (Additional file 1: Fig. S3). Thus, the *Mibin* genes must go back at least to the origin of the first cyclorrhaphan flies, perhaps about 110 million years [32]. Importantly, we could not find *Mibin* homologs in any of the species that have *Ibin* genes (Fig. 1A), which led us to hypothesize that the original *Ibin* gene has evolved directly from *Mibin* in an early ancestor of the subgenus *Sophophora*, about 55 million years ago [33]. Later, a duplication of the ancestral *Ibin* gene produced the paralogs *IbinA* and *IbinB*, of which only *IbinA* remains in the original location at 54D4, while *IbinB* has translocated to 90C1 (chromosome 3). We identified separate *IbinA* and *IbinB* genes in most members of the *Drosophila* oriental subgroups, but not in other subgroups (Fig. 1A), suggesting that the duplication happened in an ancestor of the oriental subgroups, probably less than 30 million years ago (Fig. 1A). It could also have happened earlier if one copy was secondarily lost, e.g., in the montium and other subgroups.

Both IbinA and IbinB are predicted with a high probability (0.998) to have a signal sequence at the N-terminus. The signal sequence is predicted to be cleaved between amino acids at positions 18 and 19, with a probability of 0.957 for IbinA and 0.951 for IbinB (Fig. 1B). After cleavage, the length of the predicted mature IbinA peptide is 23 amino acids; the IbinB peptide is 24 amino acids long. Mibins also have predicted signal sequences for export, but these peptides may also be subject to additional processing. In these peptides, the signal peptidase cleavage site is often followed by one or more dipeptides of the general format Xxx-Pro (XP), which are potential substrates for dipeptidyl peptidase IV-like enzymes (Fig. 1B). Of 144 investigated Mibin sequences, 102 had at least one N-terminal XP dipeptide (most commonly EP), and 13 of them had tandem repeats of 2–3 XP dipeptides (Additional file 1: Fig. S3). Similar amino-terminal dipeptides have been found in many insect antimicrobial peptides (AMPs), like cecropin, diptericin, drosocin, and hymenoptaecin, as well as in insect venom peptides [34–39]. For cecropin and the bee venom Melittin, the N-terminal dipeptides have to be removed in order to activate the peptides [34, 40]. Despite their proposed relationship, the mature Mibins have no significant sequence similarity to IbinA or IbinB, beyond the fact that several proline and glycine residues are present in each of these peptides (Fig. 1B). The Ibin family members are characterized by a conserved KNHEEWXG motif at the N-terminus (Additional file 1: Fig. S1). In contrast, the Mibin peptides share a C-terminal proline-rich motif, RPXTLPPN/QRPXXPN/DF (Additional file 1: Fig. S3), which has no counterpart in the Ibin peptides.

*IbinA* expression was previously shown to be strongly induced by bacterial infection in response to the Imd and Toll signaling pathways [21]. We have now extended these studies and compared the responses of *IbinA* and *IbinB* expression in selected conditions by quantitative reverse transcription polymerase chain reaction (RT-qPCR). We assayed the response to *Enterobacter cloacae*, which is a strong inducer of the Imd pathway, and *Micrococcus luteus*, which activates Toll signaling. The response was studied in *D. melanogaster* wild-type flies compared to *C564* > *Relish*^*IR*^, *Relish*^*E20*^ null mutant flies (with impaired Imd pathway signaling), or to *C564* > *MyD88*^*IR*^ flies with *MyD88* knockdown in the fat body (impaired Toll pathway signaling). Expression of both *IbinA* and *IbinB* is induced by both bacteria, but the inducibility of *IbinA* (Fig. 1C, D) (compared to baseline) is stronger than that of *IbinB* (Fig. 1E, F), especially with *M. luteus* infection (Fig. 1D & F). Of note, although our selected control gene *NADH dehydrogenase (ubiquinone) 39-kDa subunit* (*ND-39)* in general is very stably expressed regardless of the infection status of the flies, we observed a relatively small decrease in *ND-39* expression from 4 h onwards after *M. luteus* infection in *D. melanogaster* (RT-qPCR threshold cycle (Ct) increasing from average of 20 in uninfected flies to 21 at 4 h and 8 h and 21.6. at 24 h post-infection). We deem that the decrease in *ND-39* expression does not affect the interpretation of the results on *IbinA* and *IbinB* expression after *M. luteus* infection.

As also shown previously [21], *IbinA* expression induced by *E. cloacae* is dependent on *Relish*, and thus on the Imd pathway (Fig. 1C), whereas when infected with *M. luteus*, *IbinA* expression depended on the *MyD88* gene in the Toll pathway (Fig. 1D). Of note, like *IbinA*, *IbinB* expression was dependent on the Imd pathway when induced by *E. cloacae* (Fig. 1E), but, unlike *IbinA* expression, *M. luteus*-induced *IbinB* expression was also dependent on the expression of Imd pathway gene *Relish* (Fig. 1F). To study this further, we analyzed the importance of known Imd pathway genes (*PGRP-LC*, *Imd,* and *FADD*) for *IbinB* expression upon *M. luteus* challenge. Silencing these genes blocked induced *IbinB* expression (Additional file 1: Fig. S4A, S4B), further supporting the idea that the Imd pathway is needed for both *E. cloacae*- and *M. luteus*-induced *IbinB* expression.

Expression kinetics of *IbinB* (Fig. 1G) resemble those of *IbinA* after exposure to *E. cloacae*, whereas expression of *IbinB* peaks earlier after *M. luteus* infection (Fig. 1H) than is seen with *IbinA* [21]. *Drosophila virilis* *Mibin* expression was also strongly induced by the abovementioned bacteria (Fig. 1I, J), giving further support to the relatedness of the *Ibin* and *Mibin* genes. These data indicate that *IbinA* and *IbinB* are conserved among *Drosophila* species, and their expression is controlled by NF-κB signaling upon microbial challenge, suggesting a role in innate immunity.

### IbinA shows variable expression in aseptic stress conditions

Previous studies have shown upregulation of the *IbinA* gene, together with a handful of AMPs and other immune-responsive genes, in the heads of female flies when they were visually exposed to parasitoid wasps [23] and in the heads of male flies when they were socially isolated [24]. This suggests that IbinA and IbinB might have stress-related functions. To study this hypothesis further, we first performed RT-qPCR on heads of flies subjected to the abovementioned conditions. This led to a similar *IbinA* induction as that published earlier (Additional file 1: Fig. S4C). This expression was highly variable between samples and at a much lower level compared with that seen in infected flies. Ebrahim and coworkers [23] also observed accelerated mating in flies visually exposed to wasps. We attempted to replicate this behavioral finding by overexpressing *IbinA* under the control of the pan-neuronal *elav-GAL4* driver in female flies. However, this overexpression did not result in accelerated mating (Additional file 1: Fig. S4D). As previous work had focused on *IbinA* expression, we looked at whether *IbinB* was also upregulated in female fly heads when they were visually exposed to parasitoid wasps, but we did not see a significant increase in *IbinB* expression (Additional file 1: Fig. S4E). Similar to the wasp exposure results, single-housed (socially isolated) male flies show a moderate increase in expression of *IbinA* in their heads (Additional file 1: Fig. S4F), replicating the results from Agrawal and colleagues [24]. To investigate whether *IbinA* is responsive to physiological stress, we studied its expression patterns under other stress conditions. *IbinA* was not significantly upregulated by heat shock (Additional file 1: Fig. S4G) or osmotic stress (Additional file 1: Fig. S4H). These data indicate that infection is the predominant inducer of *IbinA* and *IbinB*.

### IbinA and IbinB do not have bactericidal or bacteriostatic properties

**Fig. 2:**
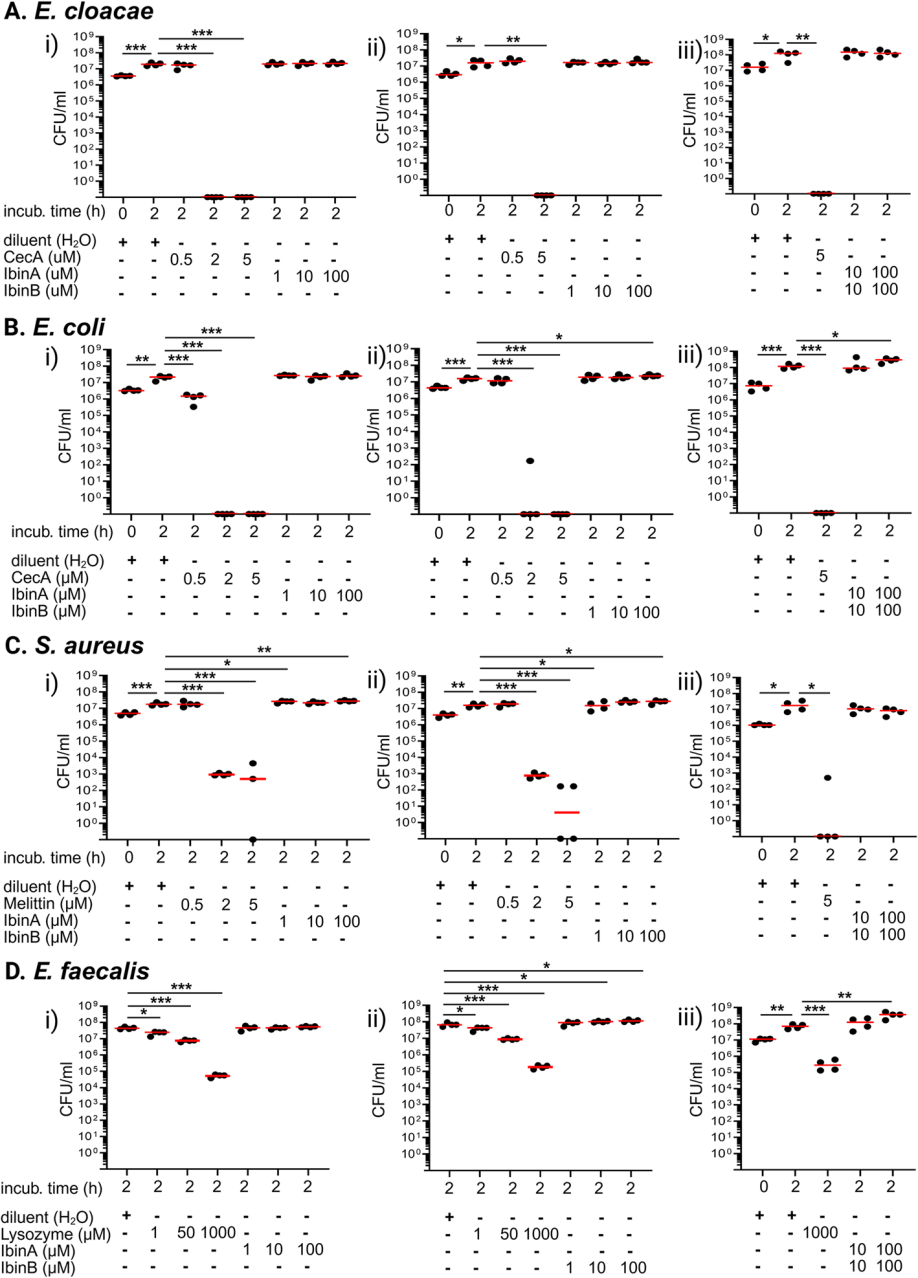
IbinA and IbinB synthetic peptides do not kill bacteria in vitro. IbinA and IbinB synthetic peptides were incubated with the following bacteria: **A** *E. cloacae*, **B** *E. coli*, **C** *S. aureus*, and **D** *E. faecalis*. The following positive controls were used: Cecropin A for *E. cloacae* and *E. coli* (**A** and **B**), Melittin for *S. aureus* (**C**), and lysozyme for *E. faecalis* (**D**). Bacteria were treated with i the IbinA peptide, ii the IbinB peptide, and iii both the IbinA and the IbinB peptides. Statistically significant differences are marked with asterisks: *p < 0.05, **p < 0.01, ***p < 0.001

While *IbinA* and *IbinB* genes are immune-inducible and the (predicted) peptides are well conserved across related species, their role in fly immunity remains unknown. As short peptides that are produced minimally in uninfected flies, and highly induced upon infection (Fig. 1C, D, E, F), IbinA and IbinB bear resemblance to AMPs and might have a function in resistance against invading pathogens. To test if IbinA and/or IbinB can prevent bacterial reproduction or directly kill bacteria, we incubated two enterobacteria (*E. cloacae* and *Escherichia coli*; Fig. 2A, B) and two cocci (*Staphylococcus aureus* and *Enterococcus faecalis*; Fig. 2C, D) with synthetic, mature IbinA (Fig. 2, panels *i*) and IbinB (Fig. 2, panels *ii*) peptides as well as the combination of IbinA and IbinB peptides (Fig. 2, panels *iii*). The AMP Cecropin A was used as a positive control for the enterobacteria, whereas Melittin (from bee venom) was used as a positive control for *S. aureus* and lysozyme (from egg white) for *Enterococcus faecalis* (Fig. 2). We did not find any effect of IbinA or IbinB peptides or their combination on bacterial growth at any concentration, in contrast to the positive controls, which were able to reduce and clear bacteria in a concentration-dependent manner (Fig. 2). These data suggest that although IbinA and IbinB are likely to have an immune response-related function, they do not act as AMPs, at least not in isolation against the four bacterial species we tested.

### IbinA and IbinB mutant flies exhibit developmental and blood cell phenotypes and changes in lifespan

**Fig. 3:**
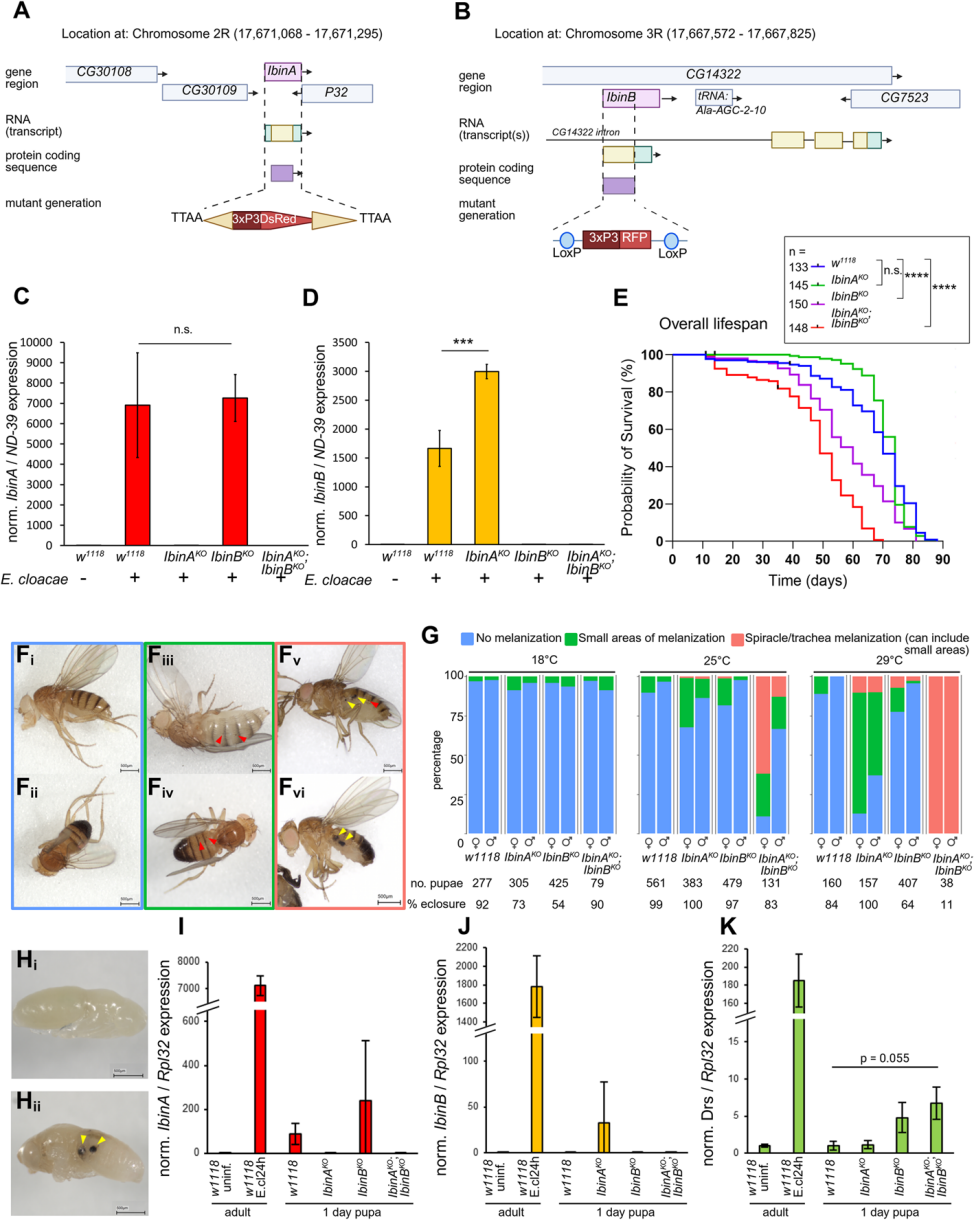
Flies lacking *IbinA*, *IbinB*, or both genes show differences in lifespan and the presence of melanized tissue in adult flies. Schematic representation of the deletion of **A** the *IbinA* gene (resulting in *IbinA*^*KO*^), with replacement by the 3xP3DsRed cassette and **B** the *IbinB* gene (resulting in *IbinB*^*KO*^), with replacement by the 3xP3RFP cassette. **C** *IbinA* expression is not detected in the *IbinA*^*KO*^ and *IbinA*^*KO*^;*IbinB*^*KO*^ lines when infected with *E. cloacae*, but it is induced normally in *IbinB*^*KO*^ flies. **D** *IbinB* expression is not detected in *IbinB*^*KO*^ or *IbinA*^*KO*^;*IbinB*^*KO*^ flies when infected with *E. cloacae*, but it is expressed more in *IbinA*^*KO*^ flies compared to wild type. **E** Lifespan of control and mutant flies. **F** Representative images of flies with melanized tissue, indicated by arrows, corresponding to categories in **G**:i female without melanized tissue, ii male without melanized tissue, iii female with small areas of melanization, iv male with small areas of melanization, v female with large melanized spots (spiracle/trachea melanization), and vi male with large melanized spots (spiracle/trachea melanization). **G** Proportions of the melanization phenotype in control and mutant lines in flies reared at 18 °C, 25 °C, and 29 °C. **H** Representative images showing i a healthy control and ii a double mutant (*IbinA*^*KO*^;*IbinB*.^*KO*^) pupa with melanized tissue. Expression of **I** *IbinA*, **J** *IbinB*, and **K** *Drs* in 1-day-old pupae of wildtype and mutant flies compared to uninfected and infected (*E. cloacae* 24 hpi) adults. **C**, **D**, **E** Statistically significant differences are marked with asterisks: *p < 0.05, **p < 0.01, ***p < 0.001, and ****p < 0.0001

To characterize the biological roles of IbinA and IbinB, knock-out mutant fly lines for the *IbinA* and *IbinB* genes were generated. Precise deletions of *IbinA* (*IbinA*^*KO*^) and *IbinB* (*IbinB*^*KO*^) genes were generated using Crispr/Cas9 technology while simultaneously knocking in a selection marker as detailed in the methods and schematically illustrated in Fig. 3A and B. Furthermore, a double mutant line for both *IbinA* and *IbinB* (*IbinA*^*KO*^; *IbinB*^*KO*^) was produced by crossing the single-mutant lines together. To validate the mutants, we confirmed that *IbinA* was not expressed in the *E. cloacae*-infected *IbinA*^*KO*^ mutant or in the double mutant (*IbinA*^*KO*^; *IbinB*^*KO*^) (Fig. 3C), and similarly, *IbinB* was not expressed in the *E. cloacae*-infected *IbinB*^*KO*^ mutant or in the double mutant (Fig. 3D). Overall lifespan showed significant differences between mutant and wild-type lines, with *IbinB*^*KO*^ and the double mutant showing shorter lifespans (Fig. 3E). *IbinA*^*KO*^ flies did not have a significantly different lifespan compared to controls (Fig. 3E). Some of the double mutant flies were also noted to have areas of melanized tissue visible through the cuticle (Fig. 3Fiii-vi: example images, compare to Fig. 3Fi and ii, wild-type flies lacking abnormal melanization, Fig. 3G: quantification). These areas of melanization frequently occurred in the abdomen at locations of spiracles and trachea (examples in Fig. 3Fv and vi, yellow arrowheads), while some individuals (both those with and without the larger spiracle/tracheal melanization spots) showed smaller melanized spots visible through the dorsal cuticle (Fig. 3Fiii and iv, red arrow heads). Melanized tissue was seen in *IbinA*^*KO*^ and *IbinB*^*KO*^ single-mutant flies much less frequently. Penetrance of this phenotype varied, with higher numbers of flies exhibiting melanized tissue when reared at 25 °C compared to 18 °C (Fig. 3G). The melanization phenotype was also found at higher rates in females compared to males (especially in *IbinA*^*KO*^*;IbinB*^*KO*^ flies at 25 °C). At 29 °C, mutant flies developed normally until emergence from the pupal case, at which point only 11% of double mutant flies eclosed, while *IbinA*^*KO*^ and *IbinB*^*KO*^ flies eclosed at much higher rates, occasionally exhibiting small areas of melanization in points under the dorsal cuticle (Fig. 3G). This was seen most often in *IbinA*^*KO*^ flies, again, particularly in females. We did not observe melanized tissue in larvae, but this phenotype was present in some individuals already at pupal stages (example images: Fig. 3H compare wild type in Fig. 3Hi to individual with melanized tissue in Fig. 3Hii). Flies with large areas of melanized tissue were sometimes observed to have brown discoloration on the dorsal side of the abdomen, putatively pericardial cells taking up melanized material (Additional file 1: Fig. S5A_i_, purple arrowhead). We also observed that male *IbinA*^*KO*^ flies frequently had areas missing from the normally expected bands of coloration on the abdomen (Additional file 1: Fig. S5A_ii_, green arrowhead), similar to the phenotype observed by Scherfer and coworkers when they knocked down *Serpin-28D*, a serpin that negatively regulates a serine protease in the melanization cascade in *D. melanogaster* [41].

As melanotic nodules are often formed by (aberrant) hemocyte activation at the larval stage [42], we checked the hemocyte profiles of *IbinA*^*KO*^ and *IbinB*^*KO*^ and the double mutant 3rd instar larvae, as well as control larvae, by classifying hemocytes as baseline immune cells (plasmatocytes, present in a healthy larva) and immune-inducible, activated hemocytes (mainly lamellocytes), based on the cell size and shape using a flow cytometer [43, 44] (Additional file 1: Fig. S5B_i-iii_). Plasmatocyte and activated hemocyte numbers in both uninfected and parasitoid wasp-infected larvae were somewhat elevated in the *IbinB*^*KO*^ and *IbinA*^*KO*^;*IbinB*^*KO*^ larvae compared to control and *IbinA*^*KO*^ larvae (Additional file 1: Fig. S5C_i-iv_). This increase could be due to enhanced release of hemocytes from sessile compartments or the lymph gland or increased proliferation in *IbinB*^*KO*^ larvae, all of which are known to occur as a response to parasitoid wasp infection [45]. However, as the increase in cell numbers in uninfected *IbinB*^*KO*^ and *IbinA*^*KO*^;*IbinB*^*KO*^ larvae is rather small and we do not observe the melanotic nodules at the larval stage, a slightly altered hemocyte profile is unlikely to be the cause of the melanotic masses in the *IbinA*^*KO*^;*IbinB*^*KO*^ flies.

Many AMPs have been shown to be upregulated during insect metamorphosis [46–49]. To see if *Ibin* genes are responding during metamorphosis, we performed RT-qPCR on approximately 1-day-old pupae from wild type and all three mutant lines. This confirmed that *IbinA* is expressed at a heightened level during the early pupal stage compared to expression in adult flies (Fig. 3I), in line with published high throughput expression data [49, 50]. We were, conversely, not able to show elevated *IbinB* expression at this stage (Fig. 3J). Our RT-qPCR results also show increased *Drs* expression in *IbinB*^*KO*^ and *IbinA*^*KO*^*;IbinB*^*KO*^ flies (Fig. 3K), suggesting possibly dysregulated humoral immunity during pupariation in mutant flies as a contributor to the melanization phenotype that we observe. The full explanation for the requirement of both *IbinA* and *IbinB* in this developmental stage requires further investigation.

### IbinA and IbinB affect susceptibility to bacterial infections

**Fig. 4:**
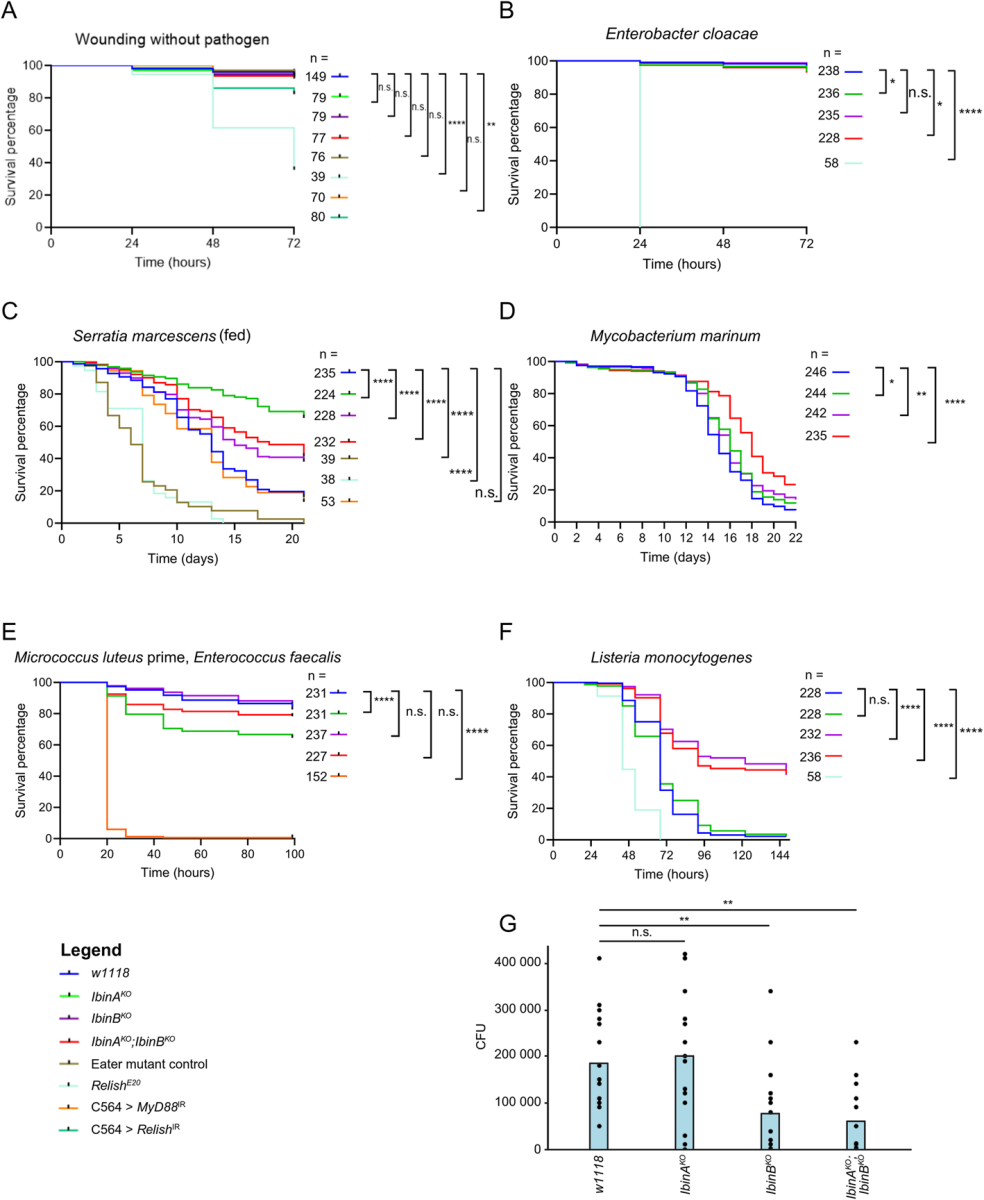
*IbinA* and *IbinB *mutants have distinct roles in immunity. **A** *IbinA* and *IbinB* mutants recover normally from wounding without a pathogen. **B** and **D**, **E**, **F** Survival of flies after infection by septic injury with selected extracellular and intracellular bacteria. **C** Survival of mutant and wild-type flies after oral infection with *S. marcescens* bacteria. **B**, **C**, **D**, **E**, **F** *Relish*.^*E20*^ (deficient in Imd pathway response), *eater* mutant (deficient in phagocytosis), and *MyD88* RNAi (deficient in Toll pathway response) flies were used as positive controls. Survival experiments are combined results from three individual experiments. **G** Bacterial loads in individual flies 36 h after *L. monocytogenes* septic injury. **A**, **B**, **C**, **D**, **E**, **F**, **G** Statistically significant differences are marked with asterisks: *p < 0.05, **p < 0.01, ***p < 0.001, ****p < 0.0001

The upregulation of *IbinA* and *IbinB* transcripts in infected flies [21] (Fig. 1) suggests a role for these peptides in the immune response, but they likely do not have a direct bactericidal role (Fig. 2). To further investigate their involvement in the immune response, infection experiments were carried out with adult male flies infected with selected bacteria. The wounding injury caused by the pin used in the infections was tolerated normally by *IbinA*^*KO*^ and *IbinB*^*KO*^ and the double mutants (Fig. 4A). Infection with *E. cloacae* showed overall very little pathogenicity, although *IbinA*^*KO*^ and *IbinA*^*KO*^;*IbinB*^*KO*^ flies showed slightly higher rates of dying (Fig. 4B). Feeding flies with the pathogen *Serratia marcescens* resulted in clear differences in survival, with *IbinA*^*KO*^ flies surviving best and all mutant lines showing improved survival compared to the wild type (Fig. 4C).

Next, we infected flies with the intracellular bacterium *Mycobacterium marinum*, which, similar to *Mycobacterium tuberculosis* in humans, infects phagocytic immune cells [51]. Double mutant (*IbinA*^*KO*^;*IbinB*^*KO*^) flies survived the *M. marinum* infection longer than the wild-type controls, and a slight improvement was seen with the single *IbinA* and *IbinB* mutants (Fig. 4D, Additional file 1: Fig. S6). To study the role of the Toll pathway, we utilized an infection method in which we first infected flies with nonpathogenic *M. luteus* in order to activate the Toll pathway, followed by a more pathogenic *E. faecalis* infection 24 h later, as described in [21, 52]. In this experiment, there was a significant reduction of survival in *IbinA*^*KO*^ flies compared with wild-type flies and the other mutant lines (Fig. 4E). We then tested *Listeria monocytogenes*, known to cause intracellular infection. Infecting flies with *L. monocytogenes* showed a clear difference in survival, with flies lacking *IbinB* showing better survival than control flies and *IbinA*^*KO*^ flies (Fig. 4F). The double mutant flies reproduced the phenotype of *IbinB*^*KO*^ individuals, being more resistant to *L. monocytogenes* infection than *IbinA*^*KO*^ flies and *w*^*1118*^ controls. Resistance to *L. monocytogenes* infection involves both the Toll pathway and Relish [53].

To gain a better understanding of the processes behind the difference in *L. monocytogenes* survival between the *IbinA* and *IbinB* mutants, we investigated whether the difference is due to resistance or tolerance to the bacteria. We infected wild-type and mutant flies with *L. monocytogenes* and homogenized the flies at 36 h post-infection. Plating the homogenates and counting colony-forming units show a significant reduction in the number of bacteria in the *IbinB-deficient* flies at this timepoint (Fig. 4G), suggesting that flies lacking *IbinB* have enhanced resistance, as they can clear bacteria more effectively or inhibit their growth. Homogenates from *IbinA*^*KO*^ flies did not show any difference compared to the wild type, in concordance with the survival results.

#### IbinA^KO^ and IbinB^KO^ have distinct effects on gene expression upon infection with L. monocytogenes

**Fig. 5:**
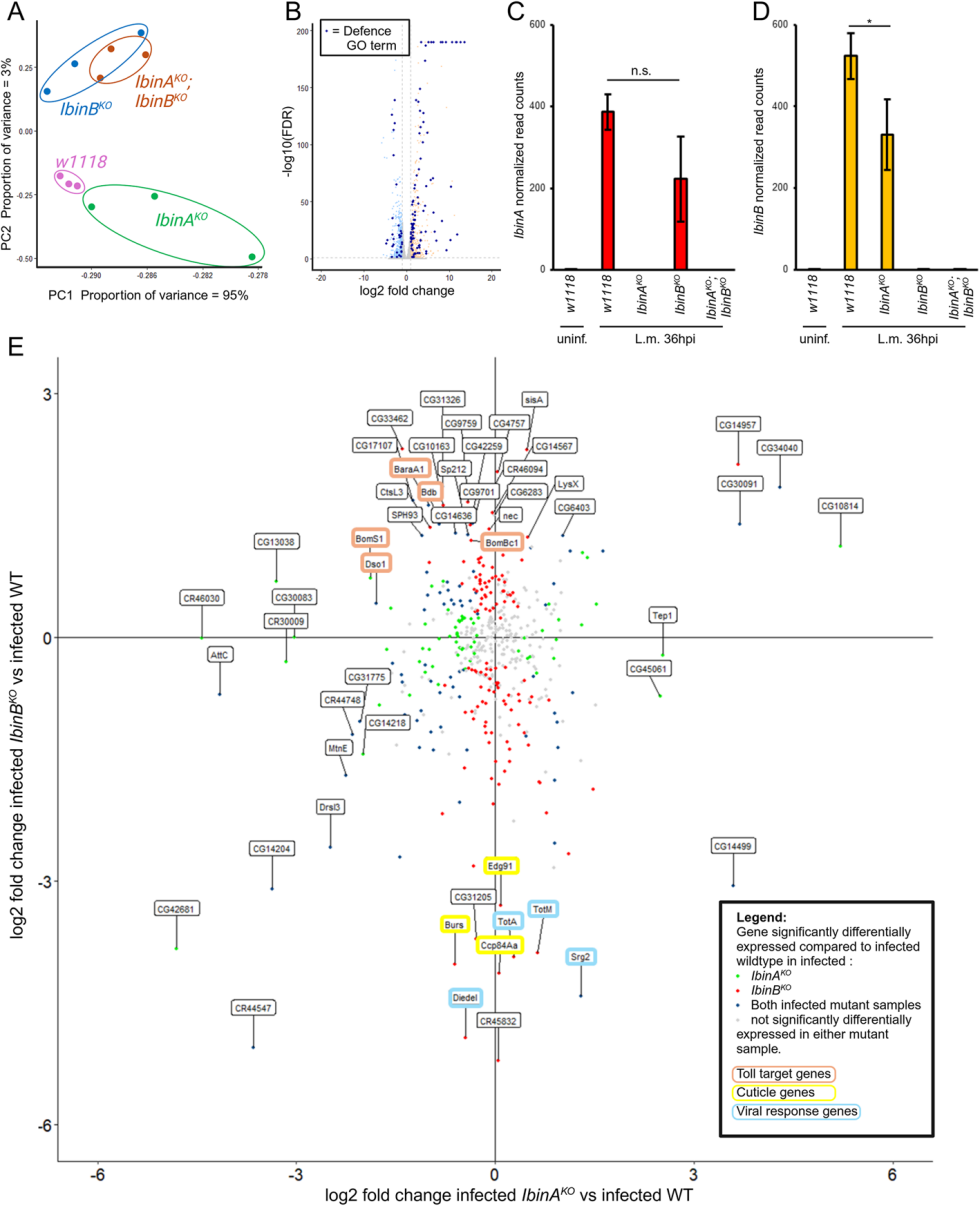
Transcriptional response to *L. monocytogenes* infection in *IbinA* and *IbinB* mutant flies. **A** PCA plot of infected samples. **B** Summary of the transcriptional response of wild-type flies to *L. monocytogenes* infection. Genes belonging to the GO term “defence response” are highlighted in blue. RNA sequencing data for **C** *IbinA* and **D** *IbinB* expression in *L. monocytogenes-infected flies are* normalized to wild-type, uninfected flies. Statistically significant differences are marked with an asterisk: *p < 0.05. **E** Genes with significantly increased expression in wild-type-infected flies vs. wild-type-uninfected flies are plotted by expression pattern in infected *IbinA*^*KO*^ (*x*-axis) or *IbinB*^*KO*^ (*y*-axis) flies compared to infected wild type. Green dots signify significantly differing expression in infected *IbinA*^*KO*^ flies compared to wild type. Red dots signify significantly differing expression in infected *IbinB*^*KO*^ flies compared to wild type. Blue dots signify significant differences in both mutant lines. Highlighted gene labels show increased expression of several Toll pathway targets in *IbinB*^*KO*^ flies and reduced expression of several viral defense and cuticle proteins in this mutant

With the aim of gaining insight into the mechanisms behind the different survival phenotypes between *IbinA* and *IbinB* mutants, we infected single and double mutants as well as wild-type controls with *L. monocytogenes* and collected samples at the 36 h timepoint for RNA sequencing. We also included uninfected samples from all four lines for comparison. Principal component analysis (PCA) of the RNA sequencing results for infected samples shows clustering of *IbinB*^*KO*^ and *IbinA*^*KO*^*;IbinB*^*KO*^ samples, separated from *w*^*1118*^ and *IbinA*^*KO*^ samples by principal component 2 (Fig. 5A), consistent with the infection survival results. PC2, however, only accounts for 3% of the total variation between samples. Additional file 1: Fig. S7A shows the PCA of uninfected samples, with all samples in close proximity along the PC1 axis, which accounts for 99% of the total variation. This fits our understanding that *IbinA* and *IbinB* genes show extremely low expression in unchallenged flies and are therefore unlikely to have significant biological effects in this context.

Lists of Gene Ontology (GO) terms for groups of differentially regulated genes between treatments were produced by filtering differentially regulated genes for up- or downregulation greater than twofold and an adjusted *P*-value less than 0.1. Using these criteria, in wild-type flies, *L. monocytogenes* infection resulted in the upregulation of 476 genes (Additional file 2: Table S2) and the downregulation of 585 genes (Additional file 2: Table S3). For upregulated genes, top GO terms included “response to biotic stimulus,” as well as “defense response” (Additional file 2: Table S4). Top GO terms from the list of downregulated genes were focused on metabolic processes (Additional file 2: Table S5). Highlighting genes in the “defense response” GO term among all differentially regulated genes shows defense response genes both up and downregulated. However, the majority of these genes are upregulated with *L. monocytogenes* infection and make up most of the most highly upregulated genes (Fig. 5B).

We began our analysis of gene expression in the mutant lines by examining *IbinA* and *IbinB* levels. Both *IbinA* and *IbinB* are highly upregulated by *Listeria* infection (Fig. 5C, D), whereas, in the mutant lines, their expression is absent in both uninfected and infected flies (Fig. 5C, D). In *Listeria*-infected flies, *IbinA* expression was, on average, lower in *IbinB*^*KO*^ flies than in wild-type controls, but this difference was not statistically significant (Fig. 5C). However, *IbinB* expression is significantly reduced in *IbinA*^*KO*^ samples compared to wild-type flies (Fig. 5D).

Following our initial analysis, we looked in detail at the differences in gene expression in the infected fly lines. To examine the differences in response to infection between the mutant lines, we plotted the 476 genes upregulated by *L. monocytogenes* infection in the wild-type flies by expression log2 fold change greater than 1 in single-mutant flies compared to the wild type (Fig. 5E). Most genes clustered around the center of the graph, signifying no difference in expression in mutants compared to the wild type. Colored dots indicate significant differences (compared to the wild type) in expression of the gene in flies lacking *IbinA* (green dots), *IbinB* (red dots), or in both cases (blue dots). Genes more highly expressed in *IbinB*^*KO*^ included several Toll pathway effectors, namely *BaraA1*, *BomS1*, *Dso1*, *Bdb*, and *BomBc1*, as well as serine proteases such as *Sp212*, *nec*, and *SPH93*. Genes with reduced expression specifically with *IbinB* absent included *Edg91*, *Burs*, and *Ccp84Aa,* all known to have roles in cuticle formation and pigmentation [54–56], and *Diedel*, *Srg2*, and the turandots *TotM* and *TotA*, with roles in viral defense and stress response.

**Fig. 6:**
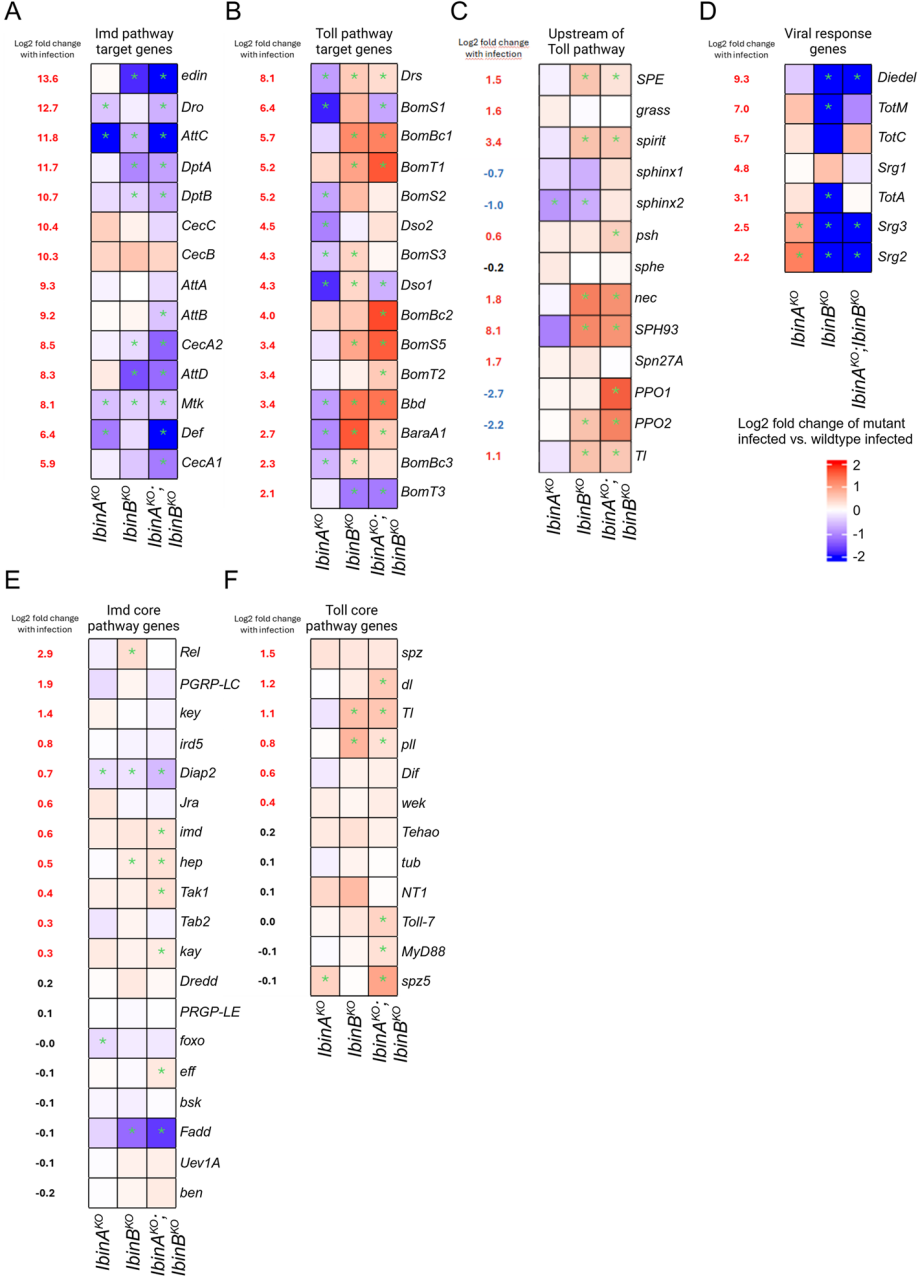
*IbinA*^*KO*^ and *IbinB*^*KO*^ flies have distinct transcriptional responses to *L. monocytogenes *infection. Heatmaps of genes selected for their known roles in the immune response. Leftmost numeric values show change in expression with infection in wildtype flies, with the following columns comparing infected mutant flies to infected wildtype. **A** Imd-responsive AMP genes. **B** Toll pathway effector genes with Log2 fold change > 2 in *L. monocytogenes*-infected wild type compared to uninfected wild-type. **C** Components of the Toll pathway upstream of the Toll receptor. **D** Genes involved in the response to viral infection with Log2 fold change > 2 in *L. monocytogenes*-infected wild type compared to uninfected wild type. **E** Imd core pathway genes. **F** Toll core pathway genes **A**, **B**, **C**, **D**, **E**, **F** Asterisks signify adjusted *p*-value < 0.05

Next, we focused on the expression patterns of selected immune-relevant genes between the mutant lines and the wild type (Fig. 6). Imd target genes [57] show little significant changes in expression in *IbinA*^*KO*^ flies, but many of these genes show significantly reduced expression in *IbinB*^*KO*^ and double mutant flies (Fig. 6A). This includes *edin*, a gene regulating hemocyte numbers during wasp infection [58], and several members of the attacin, cecropin, and diptericin families. In contrast, several Toll pathway-regulated genes [57, 59, 60] show significant downregulation in *IbinA*^*KO*^ flies and significant upregulation with *IbinB*^*KO*^ and in double mutant flies (Fig. 6B). Genes regulated in this way include *Drs*, *Bbd*, and *BaraA1*. Several other genes (including many Bomanins) showed the same trends in expression but lacking statistical significance in either *IbinA*^*KO*^ or *IbinB*^*KO*^ flies. We confirmed this pattern by RT-qPCR of *Drs* expression at 12 and 36 h timepoints after *L. monocytogenes* infection, showing that *Drs* expression is particularly highly elevated in flies lacking *IbinB* at 36 h after infection (Additional file 1: Fig. S8A).

Expression analysis of genes upstream of the Toll receptor in the Toll pathway, and genes in the melanization cascade, shows a similar pattern to that seen in Toll pathway effectors (Fig. 6C). This list includes several serine proteases, many of which are also upregulated with infection. The serine proteases *SPE*, *spirit*, *psh*, and *SPH93* all show increased expression in *IbinB*^*KO*^ and double mutant flies. The Toll receptor itself, the negative regulator *nec*, and *PPO1* and *PPO2* also show this pattern. In conclusion, these data suggest that IbinA and IbinB have distinct effects on Toll pathway target gene expression upon a challenge with the intracellular pathogen *L. monocytogenes*.

As some genes contributing to viral defense were specifically downregulated in *IbinB*^*KO*^ flies (Fig. 5E) and may also affect defense against intracellular bacteria, we took a closer look at their expression (Fig. 6D). Sting-regulated genes *Srg2* and *Srg3* show a clear reduction in expression in *IbinB*^*KO*^ and double mutant flies, whereas they have increased expression in *IbinA*^*KO*^ flies. Turandots *TotM*, *TotC*, and *TotA* are all highly upregulated during *L. monocytogenes* infection in our RNA sequencing data (Fig. 6D). *TotM* and *TotA* both show significantly reduced expression in *IbinB*^*KO*^ flies compared to the wild type. We do not see this pattern in *IbinA*^*KO*^*;IbinB*^*KO*^ flies (Fig. 6D), making turandots unlikely to be key contributors to the survival changes we see in *Listeria*-infected flies. Of note, *Diedel* shows a very clear reduction in expression in *IbinB*^*KO*^ and double mutant flies. Previous work has identified *Diedel* as a negative regulator of immunity and specifically of the Imd pathway [61]. To this end, we investigated a possible role for *Diedel* in septic *L. monocytogenes* infection. However, *Diedel* mutant flies were highly susceptible to *L. monocytogenes* infection (Additional file 1: Fig. S8C), and thus, *Diedel* does not explain the increased resistance of *IbinB* mutants.

In addition, we looked at genes central to the Imd (Fig. 6E) and Toll (Fig. 6F) signaling pathways and scavenger-like receptors (Additional file 1: Fig. S7D). Imd pathway core components showed no clear pattern, likely reflecting their generally stable expression, including during an immune response. Toll pathway core genes (Fig. 6F) showed a pattern similar to the pattern observed in genes in the pathway upstream of the Toll receptor (Fig. 6C), and in the Toll pathway targets (Fig. 6B), with increased expression of some genes in *IbinB*^*KO*^ and double mutant lines. Our list of scavenger-like receptors does not show a clearly identifiable pattern across the mutants (Additional file 1: Fig. S7D). Interestingly, *eater* expression is highly elevated in *IbinB*^*KO*^ and double mutant flies. We verified this pattern of *eater* expression in *Listeria*-infected flies at 12 and 36 h timepoints (Additional file 1: Fig. S8B). *Eater* mutant flies show decreased survival time after *Listeria* infection (Additional file 1: Fig. S8D), and thus, this change in *eater* expression may, in part, explain the enhanced resistance of *IbinB*^*KO*^ and double mutant flies.

To investigate the possible involvement of *Ibin* genes in regulating viral infection-responsive genes, we examined the expression of genes responsive to 2′3′-cGAMP injection (signifying STING pathway regulation), using lists of genes from the work of Hédelin and colleagues [62] (Additional file 1: Fig. S7B, C). *L. monocytogenes* infection results in similar regulation of many of these genes, i.e., genes that are upregulated after 2′3′-cGAMP injection are also upregulated in response to *L. monocytogenes* infection (Additional file 1: Fig. S7B) and vice versa for downregulated genes (Additional file 1: Fig. S7C). Many genes upregulated by 2′3′-cGAMP injection show increased expression in *IbinA*^*KO*^ flies and reduced expression in flies lacking *IbinB* (Additional file 1: Fig. S7B).

We investigated whether the changes in immune gene regulation with *L. monocytogenes* infection is observed in uninfected flies. GO term enrichment analysis of genes differentially regulated in uninfected *IbinA*^*KO*^ flies compared to controls suggested changes in expression of genes involved in reproduction, while the comparison between *IbinB*^*KO*^ flies and controls highlighted genes involved in metabolic processes (Additional file 2: Table S6). Immune-related terms were not enriched in either list. We also examined the expression of the immune genes highlighted in Fig. 6 in uninfected flies (Additional file 2: Table S7). Several Toll pathway-regulated genes are expressed at higher levels in mutant lines compared to controls. This includes *Drs*, *BomBc2*, and *BomS2*. This uninduced expression is, however, not comparable to the expression values observed when flies are infected (Additional file 2: Table S7).

Overall, our RNA sequencing analysis shows that *IbinB*^*KO*^ and double mutant flies have increased expression of Toll-regulated genes, including genes that regulate Toll pathway activity. Increased expression of Toll pathway target genes may explain, in part, the enhanced resistance of *IbinB* mutants to *L. monocytogenes* infection. Based on the transcriptional analysis, we conclude that the IbinB peptide functions in downregulating Toll pathway-regulated genes, including those encoding effector molecules. However, the precise point in the Toll pathway at which *IbinB* acts remains to be determined.

### IbinB downregulates Toll pathway genes when flies are infected with fungal pathogens

**Fig. 7:**
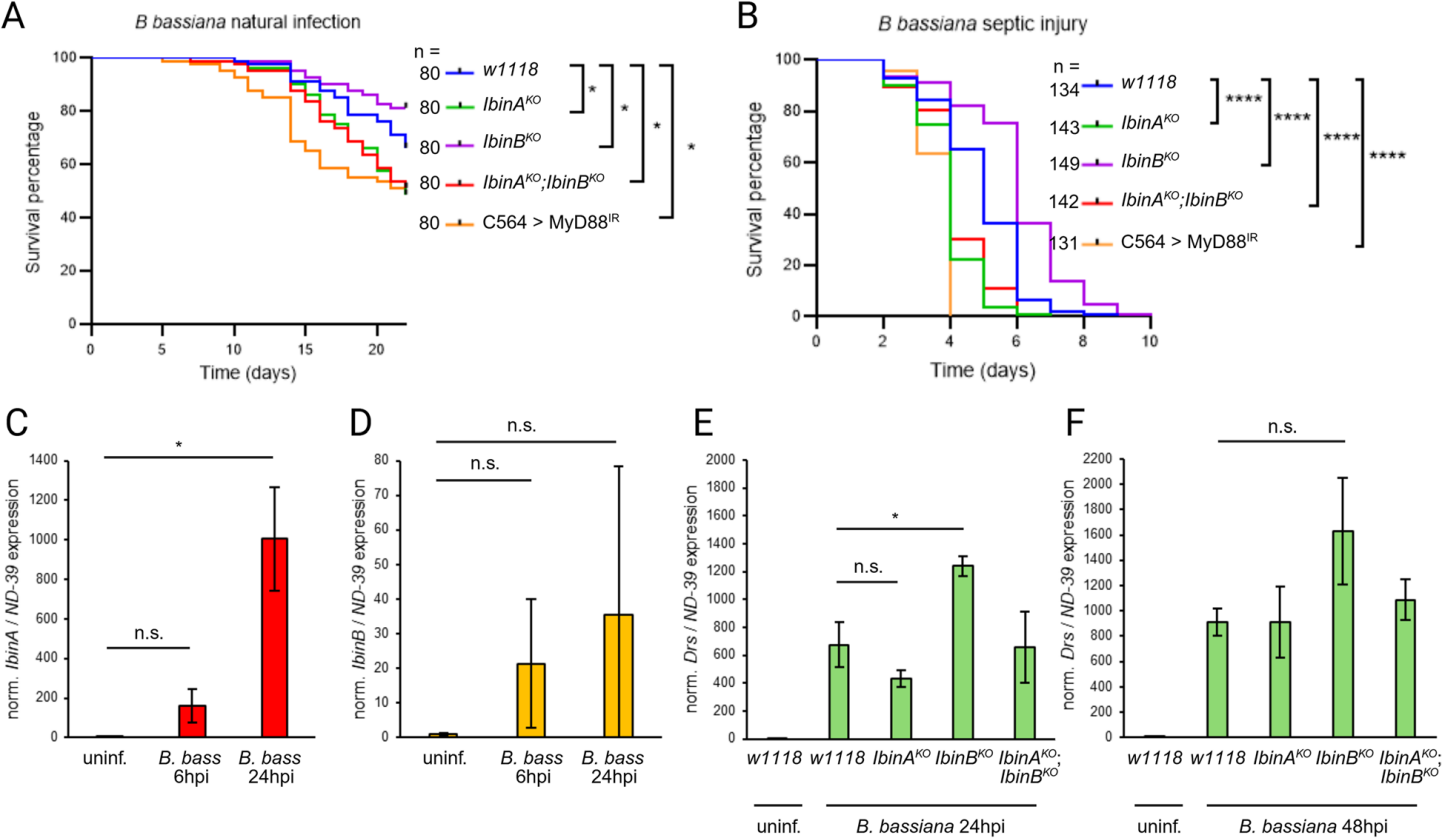
*IbinB* mutants have better survival against fungal infection and higher *Drosomycin* expression. Survival of wild-type and mutant fly lines following infection with *B. bassiana *R444 **A** through the cuticle (so-called natural infection) or **B** via septic injury. Flies with *MyD88* knockdown in the fat body (*C564* > *MyD88*^*IR*^) were used as Toll pathway-deficient positive controls. **C** *IbinA* and **D** *IbinB* expression in wild-type flies following septic injury with *B. bassiana*. *Drosomycin* expression was measured **E** 24 h and **F** 48 h after septic injury with *B. bassiana* in wild-type flies and *IbinA*^*KO*^, *IbinB*^*KO*^, and double mutant flies. **A**, **B**, **C**, **D**, **E**, **F** Statistically significant differences are marked with asterisks: *p < 0.05, **p < 0.01, ***p < 0.001, ****p < 0.0001

To study further the role of IbinA and IbinB in Toll pathway-mediated immunity, we performed fungal infection experiments, due to the central role of the Toll pathway in resistance to these pathogens [63]. We carried out infections with *Beauveria bassiana*, both through the cuticle (“natural infection”) and as systemic infections by inoculation of the fly thorax using a sharpened tungsten pin. Infections with *B. bassiana* *R444* show *IbinB*^*KO*^ flies surviving significantly better than wild-type flies. *IbinA*^*KO*^ flies, in contrast, were more susceptible. This is the case with both natural (Fig. 7A) and septic infection experiments (Fig. 7B). RT-qPCR confirmed that both *IbinA* (Fig. 7C) and *IbinB* (Fig. 7D) are expressed when flies are infected with *B. bassiana*, although *IbinB* upregulation showed a high degree of variation between samples, and therefore did not reach statistical significance. We then measured *Drs* expression at 24 (Fig. 7E) and 48 (Fig. 7F) h post-infection with *B. bassiana*, confirming that also with this infection, *IbinB* mutants exhibit significantly higher Toll pathway target gene expression than wild-type flies. These results support our finding that *IbinA* and *IbinB* expression affects Toll pathway target gene expression, and that the *Drs* expression level is correlated to survival rate.

## Discussion

Our previous study identified *IbinA* and *IbinB* as among the most highly upregulated immune-responsive genes in *Drosophila* in a wide range of infection contexts (bacterial infections as well as wasp parasitization) [21]. We add to the range of bacterial pathogens studied and show that fungal infection also triggers the expression of both *IbinA* and *IbinB*. In this work, we establish these two genes as being evolutionarily conserved among related fly species, suggesting an important and established role in the host immune response in these insects. Despite their sequence similarity, our infection results and RNA sequencing data highlight differing roles for these peptides.

The Ibin peptides appear to be unique to the subgenus *Sophophora*. Such evolutionary novelties are not rare among genes involved in immunity, and their origin is an important question. The replacement of the more ancestral *Mibin* with *Ibin* suggests a sudden shift in function of the gene in the ancestor of all sophophorans, perhaps because of a corresponding change in the target of these peptides. This brings us to the possible origin of *Mibin* in the first place. We consider it noteworthy that the *Ibin* genes are generally located near the *diptericin*/*attacin* loci in the genome of *D. melanogaster*. Many attacins and diptericins have a proline-rich N-terminal domain, and it has been suggested that this domain may have a common origin with proline-rich AMPs such as Drosocin and perhaps also Metchnikowin [36]. This idea has been further extended to include the Ibins as well as other proline-rich peptides [64]. In calyptrate flies, such as *Lucilia*, *Cochliomyia*, and *Sarcophaga*, the *Mibin* and *Attacin A*-like genes are even situated just next to each other (Additional file 1: Fig. S2).

*IbinA* and *IbinB* share a transcriptional pattern that is common among immune-regulated genes, with extremely low baseline levels in uninfected flies, apart from the elevated expression observed during pupariation. These genes show upregulation of several hundred- or thousand-fold upon infection (with *IbinA* and *IbinB* among the most highly upregulated genes during infection). Most genes with such transcription patterns are examples of the classic AMPs, such as cecropins, attacins, and diptericins, normally described as Imd-regulated, and the generally Toll pathway-regulated Drosomycin and bomanins. As effector molecules, large quantities of these peptides are produced to combat infection. Similarly regulated genes also include cytokines such as the *Unpaired* (*Upd*) genes and several serine proteases and serine protease homologues, many of which have known roles in the Toll and melanization pathways, with others having unknown or poorly defined roles in the immune response [65]. Stress-related genes, including the *turandots*, are also frequently among the most highly upregulated genes with infection. Rommelaere and coworkers [66] recently presented evidence that turandots play a tissue protective role in the context of the immune response.

Our results provide insight into where *IbinA* and *IbinB* fit within this transcriptional context. We did not find any direct bactericidal or bacteriostatic effect of Ibin peptides, pointing away from roles as AMPs. We also did not find any increased expression after heat shock. We replicated previous results showing upregulation of *IbinA* in certain stress conditions (visual exposure to parasitoid wasps and social isolation of male flies) [23, 24]. This upregulation is variable between individuals and is at a much lower level than in the case of infection. In summary, there is some evidence of a role for *IbinA* (as with other immune induced genes) in the transcriptional response to some stress conditions. However, the physiological consequences of this response require further investigation.

While Ibin peptides do not have significant roles as AMPs or in stress responses, our results do suggest an immune regulatory role for *IbinA* and *IbinB*. The RNA sequencing results show that during *L. monocytogenes* infection, flies lacking *IbinB* had increased expression of Toll pathway-mediated effectors, including most bomanins and *Drs*. In contrast, *IbinA*^*KO*^ flies had reduced expression of many of these genes. This fits the pattern we observed across infection experiments, with *IbinB*^*KO*^ flies surviving longer in infections where the Toll pathway plays a significant role in the immune response, such as *E. faecalis* infection, as well as infection with fungal pathogens. Both Toll and Imd pathways have been shown to be important for survival after *L. monocytogenes* infection [53]. These infection and transcriptome results lead us to suggest that IbinB acts as a negative regulator of Toll pathway target gene expression, with IbinA having the opposite role. The overall lifespan of the mutant lines also supports this conclusion; flies lacking *IbinB* have a shorter overall lifespan, which could be due to overactivation of the Toll pathway causing tissue damage. While a more robust Toll pathway response increases the ability to clear many pathogens, immune dysregulation has detrimental effects throughout the animal’s lifespan. We also show a dependence of *IbinB* expression on the Imd pathway, including when flies are infected with bacteria expected to trigger a Toll pathway-mediated response. This finding remains to be explained, perhaps providing evidence for crosstalk and mutual regulation between Toll and Imd pathways.

The STING pathway has been shown to be involved in the response to *L. monocytogenes* infection in *D. melanogaster* [67]. In our results, many STING-/cGAS-regulated genes are differentially regulated in flies lacking *IbinB* and in most cases are downregulated. Overall, these RNA sequencing results suggest a role for *IbinB* in modulating different aspects of the immune response, increasing the antiviral transcriptional response and reducing the expression of the Toll-responsive effector genes.

The infection survival results and transcriptome data include findings that cannot be directly explained by regulation of the Toll pathway. During *S. marcescens* infection, *IbinA*^*KO*^ flies exhibit the best survival among all lines, followed by *IbinA*^*KO*^*;IbinB*^*KO*^ and *IbinB*^*KO*^. The Toll pathway does not play a significant role in resistance to this bacterial species, suggesting that a different explanation is needed for these results. Scavenger-like receptors were differentially regulated in *Ibin* mutants, in particular, *eater*, which showed significantly increased expression in *IbinB*^*KO*^ and *IbinA*^*KO*^*;IbinB*^*KO*^ flies. This perhaps suggests changes in the number or activation state of hemocytes in flies lacking *Ibin* genes. This is consistent with our findings of changes in hemocyte number and activation state in the larvae of these mutant lines, although how the larval hemocyte state translates to immunity in adults is not clear. The cellular immune response has previously been shown to be significant in resistance to *S. marcescens* in *Drosophila* [68].

The blackened tissue we observed in adult mutant flies, likely around parts of the tracheal system of the flies, indicates that in the pupal immune environment, Ibin peptides play a protective role, either by directly protecting the tissue from attack by AMPs (as reported for turandots [66]) or by regulating the melanization response. Unlike turandots, however, we were not able to show upregulation of *Ibin* genes in response to heat or osmotic stress.

Tang and coworkers [69] observed melanization of the trachea of flies (already in larval stages) lacking the serpin Spn77Ba. This melanization led to local and systemic expression of *Drs* via the Toll pathway. The melanization we observed appears during pupariation and early adulthood, and it generally does not cover the whole trachea. However, the melanization phenotype, combined with the increased expression of Toll pathway targets, including *Drs*, points towards a role for IbinA and IbinB in the regulation of serine protease activity and thus both melanization and Toll pathway activity. The process that occurs at pupariation that leads to aberrant melanization in the mutant flies remains to be explored, as does the reason for the increased penetrance of the melanization phenotype as rearing temperature increases, and the larger melanotic spots observed on the trachea, predominantly in female flies. Flies express many AMPs at pupariation, suggesting that negative regulation of the immune system might be particularly important at this stage to prevent either aberrant melanization or tissue damage caused by humoral immune effectors. Our data suggest that *Ibin* genes are mainly expressed during pupariation and when flies are infected, providing further evidence that they are required to modulate the humoral immune response and/or melanization under specific circumstances. Scherfer et al. [41] also showed some melanization of the trachea in flies lacking proper negative regulation of melanization, suggesting that the trachea is particularly susceptible to aberrant melanization. They speculate that this is due to oxygen being a substrate for phenoloxidase [41].

## Conclusions

Overall, our results uncover roles for IbinA and IbinB in regulating the immune response. Specifically, IbinB has a negative regulatory effect on the expression of Toll pathway effector genes, while IbinA, in the context of infection, appears to play the opposite role. In the case of *L. monocytogenes* infection, as well as in resistance to *E. faecalis* and fungal pathogens, *IbinB* mutants are able to mount a more effective response, likely due to their higher expression of Toll effectors, including *Drs*. While *IbinB*^*KO*^ flies consistently survive better than wild-type individuals in the context of bacterial infection, these flies have overall shorter lifespans, which is further evidence for the role of *IbinB* in regulating the immune system, thus preventing excessive tissue damage from aberrant immune activation. Observations of melanization during pupariation further suggest an immune regulatory and tissue protective role for *Ibin* genes. Our findings on the roles of *Ibin* genes in immune regulation highlight the importance of appropriate levels of immune activation, both during immune challenge and in unchallenged conditions throughout development.

## Methods

### Bioinformatic analysis of IbinA, IbinB, and Mibin genes

*Ibin* homologs were initially identified by simple blastp and tblastn searches of the *refseq_protein* and *refseq_genomes* databases, followed up by repeated searches with some of the initially retrieved hits, and similar searches of the *wgs* database for selected taxa. To limit the scope of the study, we have refrained from expanding the set as new species have been added to the databases. A consensus phylogenetic tree, showing the occurrence and chromosomal locations of *IbinA*, *IbinB*, and *Mibin* orthologs, was built from [33, 70, 71], with time calibration from [33]. The first *Mibin* homologs were identified by inspecting all open reading frames in the 1-kb intergenic region between *Drosophila busckii* homologs of *D. melanogaster* genes *CG30109* and *P32*, where *IbinA* is located. Further, *Mibin* homologs were then identified by tblastn searches of the *refseq_protein*, *refseq_genomes*, and *TSA* databases. Multiple sequence alignments were done by Clustal Omega at the The EMBL-EBI Job Dispatcher (https://www.ebi.ac.uk/jdispatcher/msa/clustalo) and further manually curated. Signal peptide sequence prediction was done using SignalP-6.0 at the DTU Health Tech server (https://services.healthtech.dtu.dk/services/SignalP-6.0).

### In vitro bacterial killing assay

The in vitro bacterial killing assay was modified from [72]. Briefly, the selected bacteria were grown overnight either in Luria–Bertani (LB) or brain–heart infusion (BHI) medium as detailed above. The next morning, bacteria were subcultured in fresh medium for 1–2 h to reach an OD600 of 0.33 (exponential growth phase). A 1:10 dilution of bacteria was added to a fresh solution of LB/BHI including the peptide tested or the antimicrobial agent control, at the desired final concentrations.

Mature IbinA and IbinB peptides were obtained from GenScript Biotech (Netherlands, B.V.) as lyophilized powders. Peptide sequences are as follows: IbinA: KNHEDWGGYRPSDYDPRPYFRQF and IbinB: KNHEEWKGQRPWDYDRRPPSNPYA. Peptides were dissolved in ultrapure H_2_O and kept at − 80 °C until use. The following antimicrobial agents were used as positive controls: Cecropin A (Sigma Aldrich, no. C6830) for *Enterobacter cloacae* and *Escherichia coli*, Melittin from honeybee venom (Sigma Aldrich, no. M2272) for *Staphylococcus aureus*, and lysozyme (EMD Millipore Corp., USA, no. 4403) for *Enterococcus faecalis*. Sterile ultrapure H_2_O was used as the negative control. Samples were incubated for 2 h at 25 °C, after which they were placed on ice. Serial dilutions of bacteria were carried out in ice-cold 1 × PBS, and 3 µl (four replicates per treatment) was plated onto LB/BHI agar. Plates were grown overnight at 29 °C. Colony-forming units (CFU/ml) were counted from the appropriate dilution on the agar plate and were calculated taking all dilution steps into account.

### Drosophila lines

The *D. melanogaster w*^*1118*^ and *Canton-S* lines were used as control lines. The *w*^*1118*^ line has been kept in the laboratories of the authors for over 30 years, and our GAL4-driver lines are backcrossed into this genetic background. The UAS/GAL4 system [73] was used for silencing selected genes in the selected tissue. To study the effect of the Toll and Imd pathways, *UAS-MyD88*^*IR*^ (GD no. 25399), *UAS-Rel*^*IR*^ (GD no. 49414), *UAS-PGRP-LC*^*IR*^ (GD no. 51968), and *UAS-Imd*^*IR*^ (GD no. 9253) RNAi lines from the Vienna Drosophila Resource Center (VDRC) and *UAS-FADD*^*IR*^ (12297R-1, III) from the NIG-Fly center in Kyoto were used. The *C564-GAL4* line, driving strong expression of the UAS construct in the fat body [74] and in some other tissues, was a gift from Prof. B. Lemaitre (Global Health Institute, Swiss Federal Institute of Technology Lausanne, Switzerland). *Relish*^*E20*^ flies (Bloomington stock no. 9457) contain a null allele of the *Relish* gene [75]. The transheterozygous *eater* mutant flies were obtained by crossing together the Bloomington Stock Center deficiency lines *Df(3R)D605* (stock no. 823) and *Df(3R)Tl-I* (stock no. 1911), as in Kocks et al. [76]. *Diedel* knockout line (generated by imprecise p-element excision described in [77]) and its background control line were a kind gift from Professor Jean-Luc Imler (CNRS, Strasbourg, France).

*IbinA* and *IbinB* knock-out (KO) mutants were generated at Wellgenetics Inc. (Taiwan, www.wellgenetics.com) via CRISPR/Cas9-mediated genome editing by homology-dependent repair (HDR). For *IbinA*^*KO*^, one guide RNA and a double-stranded deoxyribonucleic acid (dsDNA) plasmid donor were used, whereas for *IbinB*^*KO*^ two guide RNAs and a dsDNA plasmid donor were used. The mutants were backcrossed to the control line used in the experiments (*w*^*1118*^). The *IbinA*^*KO*^;*IbinB*^*KO*^ double mutant was generated by crossing the backcrossed mutant lines together to obtain the genotype as follows: *w*^*1118*^; *IbinA*^*KO*^;*IbinB*^*KO*^.

### Scoring and imaging the pupal and adult melanization phenotypes

To score melanization phenotypes, 10 mated female flies were allowed to lay eggs in a vial at 25 °C for 24 h, before they were discarded. Vials were then kept at the experimental temperature. When eclosion began, each individual fly was visually inspected using a dissecting microscope and was scored into one of three categories: no melanization visible, small areas of melanization, or spiracle/trachea melanization (flies were included in this category if they featured both spiracle/trachea melanization, as well as smaller areas visible through the dorsal cuticle). This was performed for 5 days from the beginning of eclosion, after which pupal cases were counted to determine the eclosion percentage. Representative images of phenotypes were taken of flies raised at 29 °C using a Nikon SMZ475T microscope.

### Larval hemocyte analysis

Mated females of the *Ibin* mutants and the control were allowed to lay eggs for 24 h at 25 °C. The eggs were then transferred to 29 °C for further development. To analyze the hemocytes after wasp infection, 2nd instar larvae were exposed to 10 female *L. boulardi* strain G486 wasps for 2 h at 22 °C, after which the wasps were removed, and the larvae were placed back at their rearing temperature until they reached mid- to late 3rd instar larval stage. Then, the larvae were gently washed by brushing in water to remove food remnants and dissected in 20 µl of 1% bovine serum albumin (BSA) in 1 × PBS. The hemocyte sample was pipetted into a microtube with 80 µl of 1% BSA in PBS. A total of 30 µl of the sample was run with a BD Accuri C6 flow cytometer (BD Biosciences). In the case of wasp-infected larvae, the presence of a wasp larva was visually confirmed prior to sample collection. Each genotype was analyzed in triplicate (3 × 10 larvae), with equal numbers of males and females. Hemocytes were first separated from the debris released when dissecting the larvae based on their location in a forward scatter area (FSC-A) — side scatter area (SSA-A) plot [78] (Additional file 1: Fig. S5Bi). Separation between inactivated “basal” plasmatocytes and activated hemocytes was made based on the protocol described [43, 44]. Shortly, plasmatocytes are small and round in their inactivated state and can be gated using the forward scatter area vs. forward scatter height plot, where round cells form a population detected at a 45° angle (Additional file 1: Fig. S5Bii). Lamellocytes are irregularly shaped, which places them outside of the 45° angle in the forward scatter area *vs.* forward scatter height plot (Additional file 1: Fig. S5Biii).

#### Culturing and processing of microbes for infections

*Micrococcus luteus* and *Enterobacter cloacae* were cultured on LB agar plates under antibiotic selection, and *Enterococcus faecalis* was cultured in BHI medium as described previously [21]. Bacteria were collected from the plate or pelleted and mixed with glycerol to create a thick paste. *Listeria monocytogenes* was grown overnight in 5-ml BHI medium, shaking at 37 °C, after which the culture was diluted 1:25 in fresh medium and grown to an OD_600_ of 0.75. Following this, 1 ml of bacterial suspension was centrifuged at 500 × g for 3 min. The supernatant was removed, and the pellet was resuspended in 100 µl of 50% glycerol. *Mycobacterium marinum* ATCC 927 was grown at 28 °C in 7H9 medium supplemented with ADC enrichment and Tween 80 to achieve an OD_600_ value of 0.75. The bacteria were prepared by centrifugation of 1 ml of bacterial culture for 3 min at 10,000 rcf, and the supernatant was removed. The pellet was resuspended in 100 µl of 50% glycerol. *Serratia marcescens* Db11 was grown at 37 °C in LB broth to an OD_600_ of 1.0. *Beauveria bassiana* strain R444 (BB-PROTEC) commercial spores were produced and provided by Andermatt Biocontrol as fungal spores mixed with talk powder.

### Fly infections and survival monitoring

Fly infections were carried out using multiple infection routes and methods. To cause septic injury, flies were pricked in the thorax area with a sharpened tungsten pin dipped in the thick bacterial paste. Infections with *Serratia marcescens* by feeding were carried out essentially as in [68]. Briefly, flies were placed in vials with cotton wool plugs soaked with 5 ml of either a 50-mM sucrose solution or the same sucrose solution inoculated with a 1:100 dilution of *S. marcescens* bacteria. For causing a natural fungal infection, 30 mg of commercial spores was added to an empty fly vial. Flies were added to the vial and tapped to the bottom of the vial for 30 s to cover flies in fungal spores, as described previously [52, 59]. For systemic fungal infections, 30 mg of commercial fungal spores was suspended in 100 µl of 50% glycerol. Flies were pricked in the thorax area with a sharpened tungsten pin dipped in the fungal paste. Depending on the rate of death from the infection, the survival of flies was recorded either several times a day, daily, or every other day. For long survival times, the food was changed twice a week until the end of the experiment.

### Bacterial load

Colony-forming units (CFU) per fly was measured in flies 36 h post-*Listeria monocytogenes* infection, as modified from [44]. Eight flies per strain were infected. To kill the cutaneous bacteria, each fly was dipped in 70% ethanol for 30 s, after which the fly was placed in 100 μl of PBS and homogenized with a plastic pestle. A tenfold dilution series was prepared in 96-well plates, and 3 μl of each dilution was plated on LB-agar plates and incubated overnight at 37 °C. Colonies were counted under a light microscope.

### RNA extraction and RT-qPCR

RNAs were extracted, and RT-qPCR was carried out as described previously [31]. Three replicates of five male flies per genotype were collected and snap-frozen on dry ice. TRI reagent (MRC, Thermo Fisher Scientific) was added to the frozen flies, and the flies were homogenized using a micropestle (Thermo Fisher Scientific). Thereafter, the total RNAs were extracted according to the manufacturer’s instructions. RNAs were dissolved in nuclease-free water, and the concentrations and purity were measured using the NanoDrop 2000 equipment (Thermo Scientific). RT-qPCR was carried out on the total RNA samples (∼40 ng/sample) with the iTaq Universal SYBR Green one-step kit (Bio-Rad, Hercules, CA, USA). A gene encoding a subunit of mitochondrial respiratory complex I, NADH dehydrogenase (ubiquinone) 39-kDa subunit (ND-39), was used as a steadily expressing control gene to normalize differences in RNA amounts between samples. The primers used are listed in Additional file 2: Table S8.

### Stress experiments

Heat shock was performed on 3- to 5-day-old male flies. Flies were maintained at 25 °C until the start of the experiment. Fly vials were transferred to a pre-heated water bath at 36 °C for 1 or 4 h. Immediately after exposure, flies were anesthetized on a CO_2_ pad and decapitated using a scalpel blade, with heads and bodies separately transferred to − 80 °C storage in preparation for RNA extraction. Osmotic stress experiment was performed by feeding flies normal fly food with 4% NaCl added. Flies were maintained on food for 8 or 24 h before flash freezing and RNA extraction. Separately, flies were maintained on 4% NaCl food with blue dye (Brilliant blue, Carbosynth) to confirm that 100% of the flies maintained on 4% NaCl food consumed the food. Food consumption was confirmed by checking for the presence of blue dye in the fly guts under a light microscope. *IbinA* and *IbinB* induction was measured by RT-qPCR in male and female Canton-S flies that were visually exposed to *L. boulardi* strain G486 wasps, as described in [23]. Male and virgin female flies at 2 to 5 days post-eclosure were housed in vials of 20 flies. Each vial was surrounded by 6 vials for 2 h, each of which contained 15 male and 15 female wasps. Immediately following the 2-h exposure, flies were decapitated as described above, and heads and carcasses were frozen at − 80 °C for RNA extraction.

For measuring courtship delay time, male and female flies were collected into separate vials within 6 h after eclosure and maintained for 2 days at 25 °C. Individual male and female flies were transferred using suction (without anaesthetization or tapping) into the separate sides of a mating chamber at 8 am. Mating chambers were kept for 1 h at 25 °C. The divider was then removed, allowing interaction of the male and female flies. Courtship and mating behaviors were filmed using a cell phone (iPhone 5S, Apple, USA) placed in the incubator above the mating chambers. Flies were allowed to interact for 1 h before the experiment ended, with time to copulation for individual pairs noted from the video recordings.

Social isolation was performed on 2-day-old adult males. Flies were transferred by suction into individual vials. Vials were kept in a 25 °C incubator for 3 days, separated by dividers to prevent visual contact between the individual flies. Individuals were then anesthetized and decapitated in preparation for RNA extraction. Gene expression levels were measured separately from heads and bodies.

### Lifespan

Three- to five-day-old adult males were used for lifespan experiments. Flies were maintained in vials of 25 individuals at 25 °C. Flies were transferred to new food vials twice per week, with new deaths recorded on days when the flies were transferred. The experiment continued until all flies were dead.

### Sample preparation for RNA sequencing

Flies were infected with *Listeria monocytogenes* using the same protocol as for infection survival experiments. Live infected flies and uninfected controls with similar incubation times were collected at the 36 h post-infection timepoint. One biological replicate consisted of five individual flies, with three biological replicates per treatment. Total RNA was extracted using the TRI reagent protocol described above.

### RNA sequencing

Quality control of isolated RNAs, preparation of the RNA library (including quality control), the whole transcriptome sequencing, and data analysis were carried out by Novogene Co. Ltd, UK (www.novogene.com). In short, messenger RNA was purified from the total RNA using poly-T oligo-attached magnetic beads. RNA was fragmented, after which the first-strand complementary deoxyribonucleic acid (cDNA) synthesis was carried out using random hexamer primers, followed by second-strand cDNA synthesis. Following library construction consisted of end repair, A-tailing, adapter ligation, size selection, amplification and purification. The quality-controlled library preparations were quantified, pooled, and sequenced using an Illumina platform. Paired-end 150-bp technology was used to sequence the samples. Sequence data analysis first included quality control, where raw reads were processed by removing reads containing adapters, reads containing poly-N, and low-quality reads from the raw data. Reads were mapped to the *D. melanogaster* reference genome (Genome ID: ensembl_drosophila_melanogaster_ bdgp6_gca_000001215_4). Gene expression levels for each gene were calculated as FPKM (fragments per kilobase million).

### Statistical analysis

Statistical analyses of RT-qPCR gene expression results were carried out using a two-tailed *t*-test for two samples assuming equal variances using R version 4.3.3 [79]. Statistical analyses of fly survival from infection and lifespan experiments were carried out using the log-rank (Mantel-Cox) test with Prism version 10.3.1 (GraphPad Software LLC). The data on hemocyte counts were analyzed with R version 4.3.3 [79], using a negative binomial generalized linear model (MASS package [80]). The least square means (estimated marginal means) were analyzed for multiple comparisons [81], and the Tukey method was used for adjusting the *p*-value. Heatmaps were generated using the ComplexHeatmaps R package version 2.18.0 [82]. Differential gene expression was calculated by Novogene using the R package DESeq2 [83], with the false discovery rate calculated using the Benjamini–Hochberg method. GO term and pathway enrichment analysis was performed using FlyMine [84].

## Supplementary Information

Additional file 1. Figures S1-S7. FigS1. Ibin open reading frames. FigS2. Chromosomal location of *Ibin* and *Mibin* genes in different species. FigS3. Mibin open reading frames. FigS4. *IbinB* expression is dependent on functional Imd pathway. *IbinA* shows varied expression upon exposure to various stressors. FigS5. Example images of *Ibin* mutant phenotypes. Flow cytometric analysis of larval hemocytes in *Ibin* mutants and controls. FigS6. Additional heatmaps of immune-relevant genes at 36hpi with *L. monocytogenes* infection in *Ibin* mutants and controls. FigS7. qPCR verification of *eater* and *Drs* expression at 36hpi with *L. monocytogenes* infection. Survival of *eater* and *Diedel* mutant flies from *L. monocytogenes* infection.

Additional file 2. Tables S1–S8. Table S1. RNA sequencing fold change comparisons. Table S2. Genes upregulated with *L.m*. infection in wildtype (*w*^*1118*^). Table S3. Genes downregulated with *L.m.* infection in wildtype (*w*^*1118*^). Table S4. Gene ontology hits from the Table S2 list. Table S5. Gene ontology hits from the Table S3 list. Table S6. Differentially expressed genes in uninfected single mutants vs w^1118^ controls, and GO enrichment from these lists. Table S7.Average normalized read counts with the standard deviation of genes shown in Fig. 6, for all (uninfected and infected) samples. Table S8. RT-qPCR primers used in this study.

## Data Availability

The transcriptome analysis dataset is available via the Gene Expression Omnibus database, using the GEO accession number GSE293484 [85].

## Abbreviations

AMP: Antimicrobial peptide
BHI: Brain-heart infusion
BSA: Bovine serum albumin
cDNA: Complementary deoxyribonucleic acid
CFU: Colony-forming units
Ct: Threshold cycle
dsDNA: Double-stranded deoxyribonucleic acid
FPKM: Fragments per kilobase million
FSC-A: Forward scatter area
GO: Gene ontology
HDR: Homology-dependent repair
KO: Knockout
LB: Luria-Bertani
PAMP: Pattern-associated molecular pattern
PBS: Phosphate-buffered saline
PCA: Principal component analysis
PGRP: Peptidoglycan recognition protein
PRR: Pattern recognition receptor
RNA: Ribonucleic acid
RT-qPCR: Real-time quantitative polymerase chain reaction
SSA-A: Side scatter area
