# Supplementary material for: IbinA and IbinB regulate the Toll pathway-mediated immune response in Drosophila melanogaster
Source: BMC Biol. 2026 Jan 9;24:33. doi: 10.1186/s12915-025-02501-7 (PMC12882233; doi:10.1186/s12915-025-02501-7)
Supplement: Supplementary file 1 — Additional file 1. Figures S1-S7. FigS1. Ibin open reading frames. FigS2. Chromosomal location of Ibin and Mibin genes in different species. FigS3. Mibin open reading frames. FigS4. IbinB expression is dependent on functional Imd pathway. IbinA shows varied expression upon exposure to various stressors. FigS5. Example images of Ibin mutant phenotypes. Flow cytometric analysis of larval hemocytes in Ibin mutants and controls. FigS6. Additional heatmaps of immune-relevant genes at 36hpi with L. monocytogenes infection in Ibin mutants and controls. FigS7. qPCR verification of eater and Drs expression at 36hpi with L. monocytogenes infection. Survival of eater and Diedel mutant flies from L. monocytogenes infection. [file 12915_2025_2501_MOESM1_ESM.pdf]

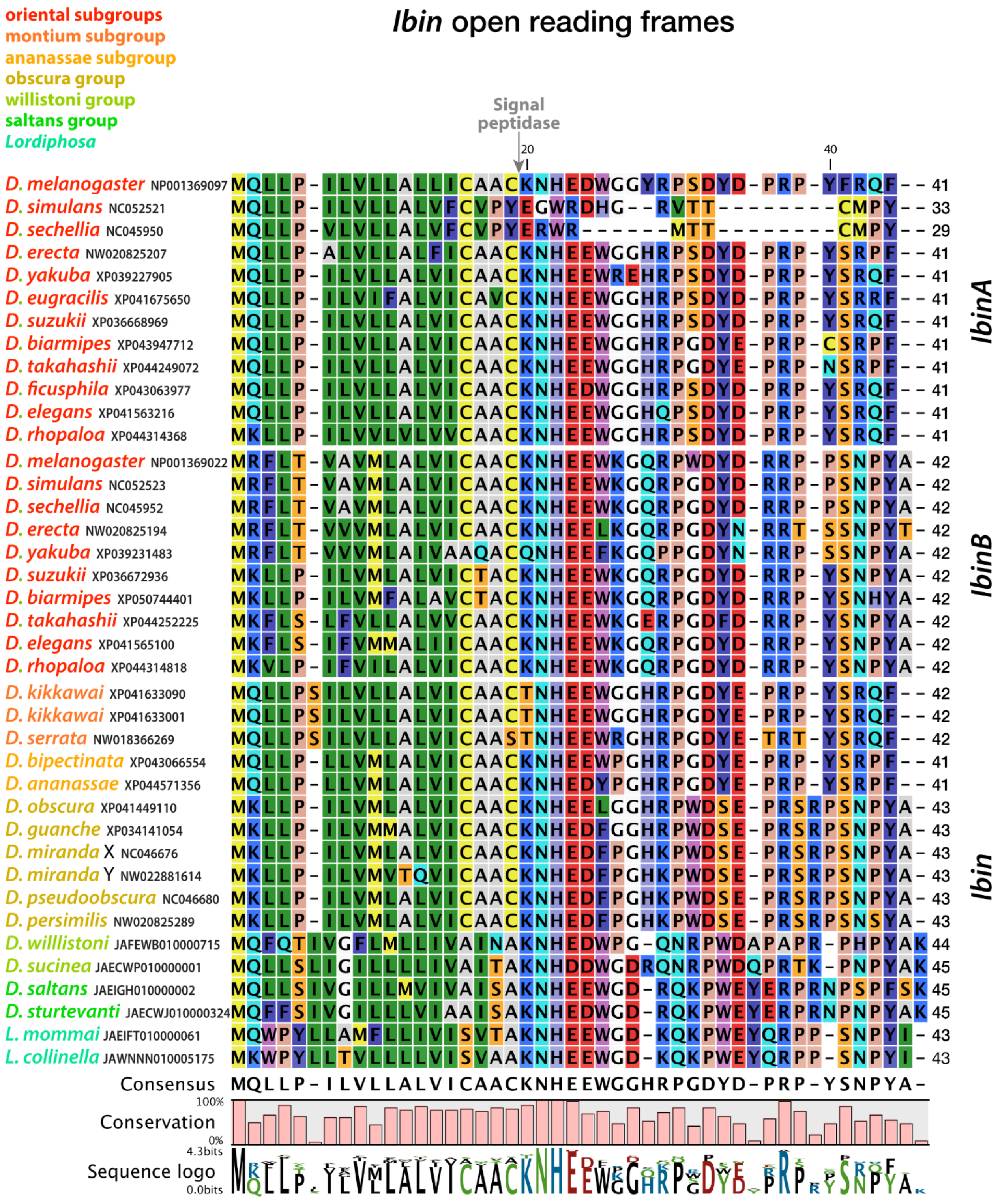

Sup. Fig. 1. Alignment of IbinA and IbinB open reading frames across fly species.

Chromosomal location of *Ibin* and *Mibin* genes in different species

1. Acalyptratae: Drosophilidae

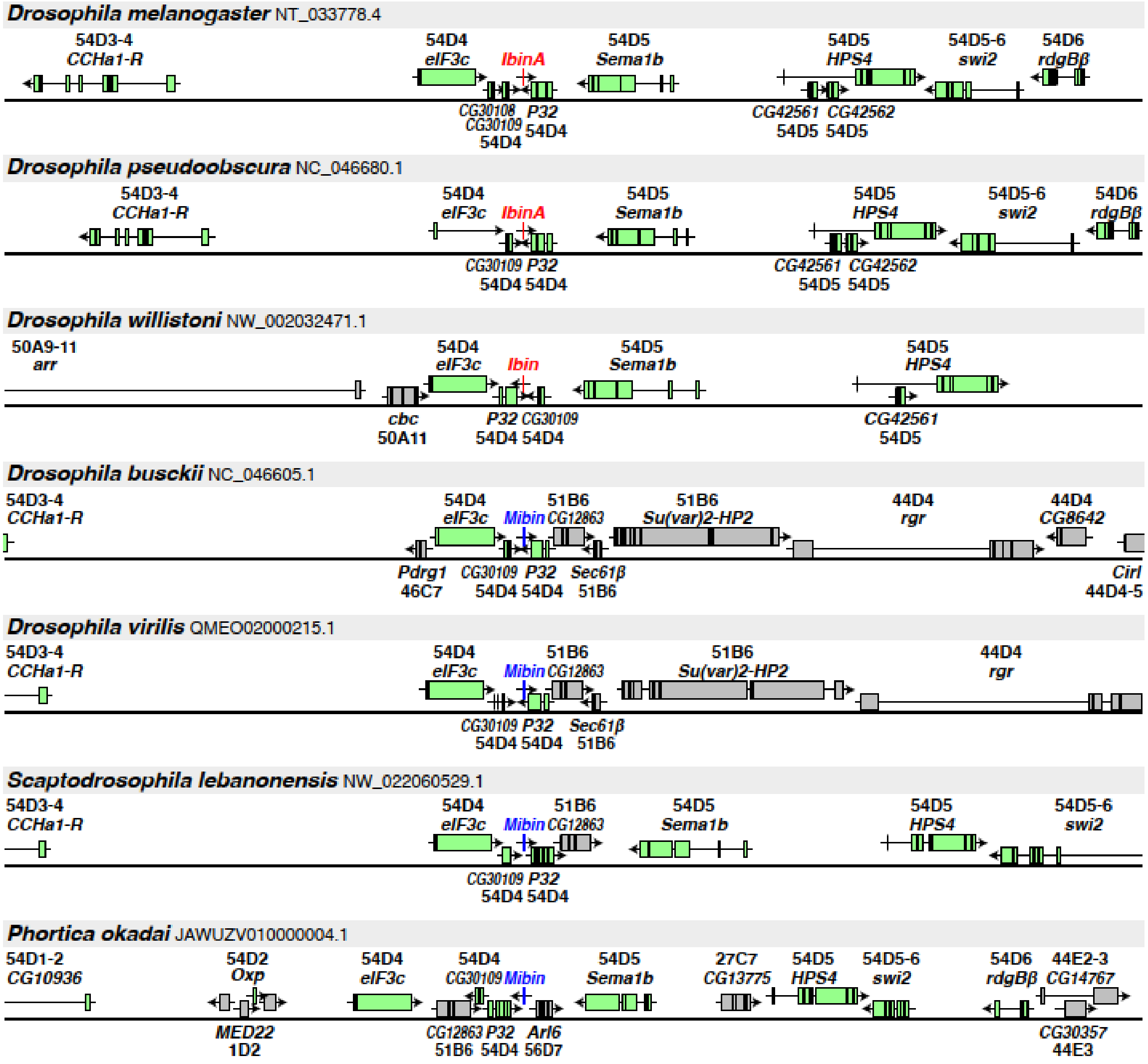

2. Acalyptratae: Diopsidae

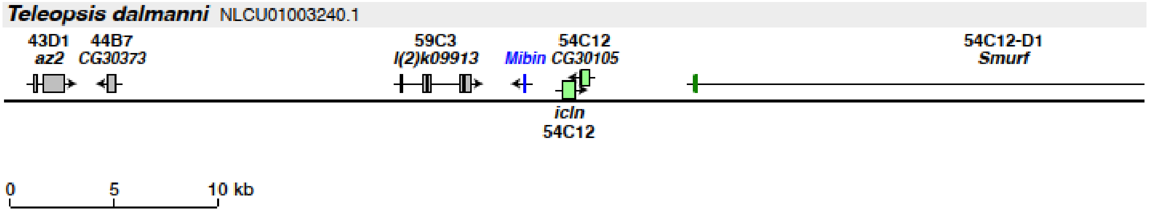

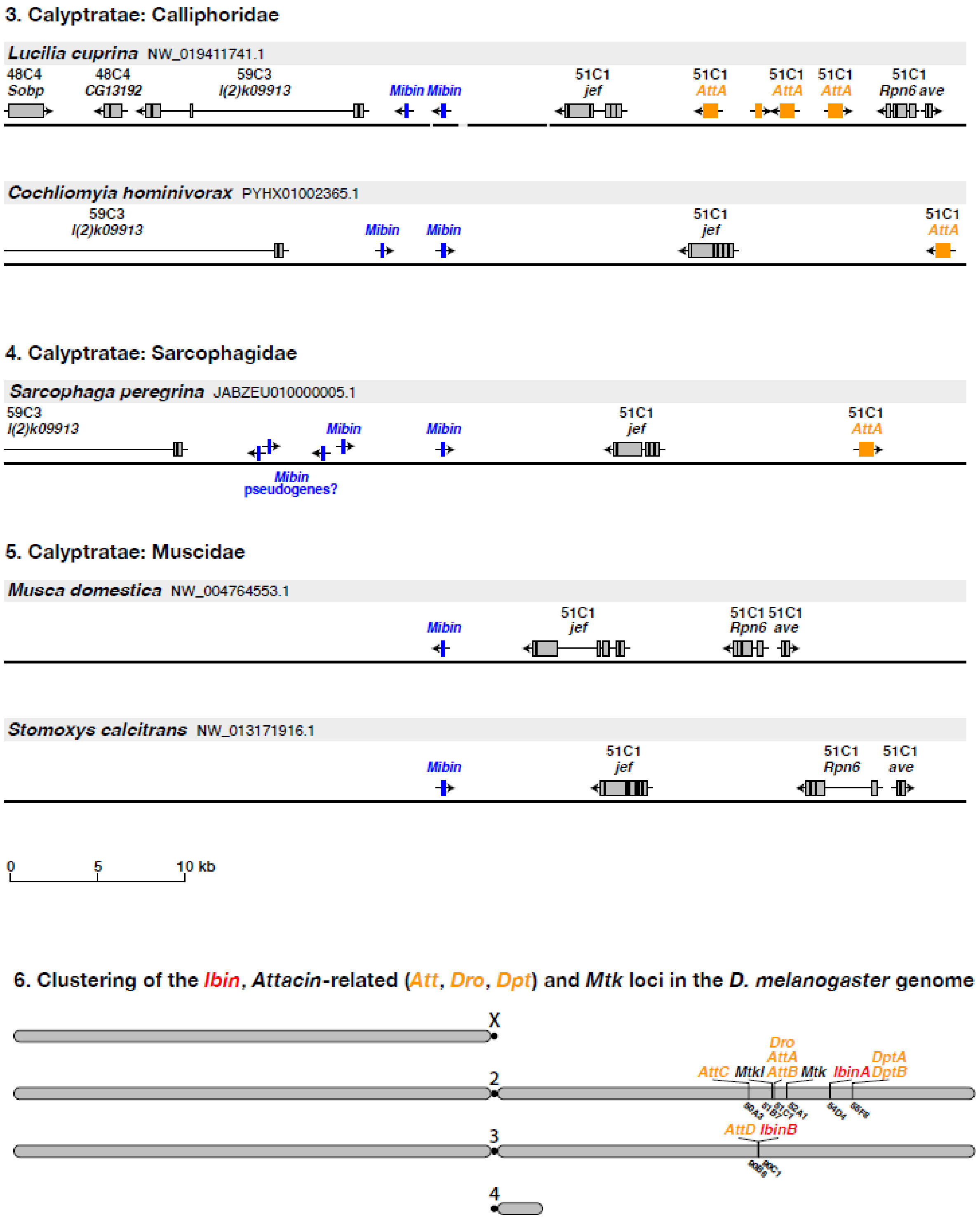

Sup. Fig. 2. Chromosomal location of *Ibin* and *Mibin* genes across species. Maps of genes around the *IbinA* and *Mibin* genes in selected acalyptrate (page 1) and calyptrate (page 2) species. For each open reading frame, the name and chromosomal location of the orthologous *D. melanogaster* gene is indicated. Orthologs of genes in the near vicinity of *IbinA* in *D. melanogaster*, at 54C12-D6, are shown in green color.

Alignment of Mibin open reading frames across fly species

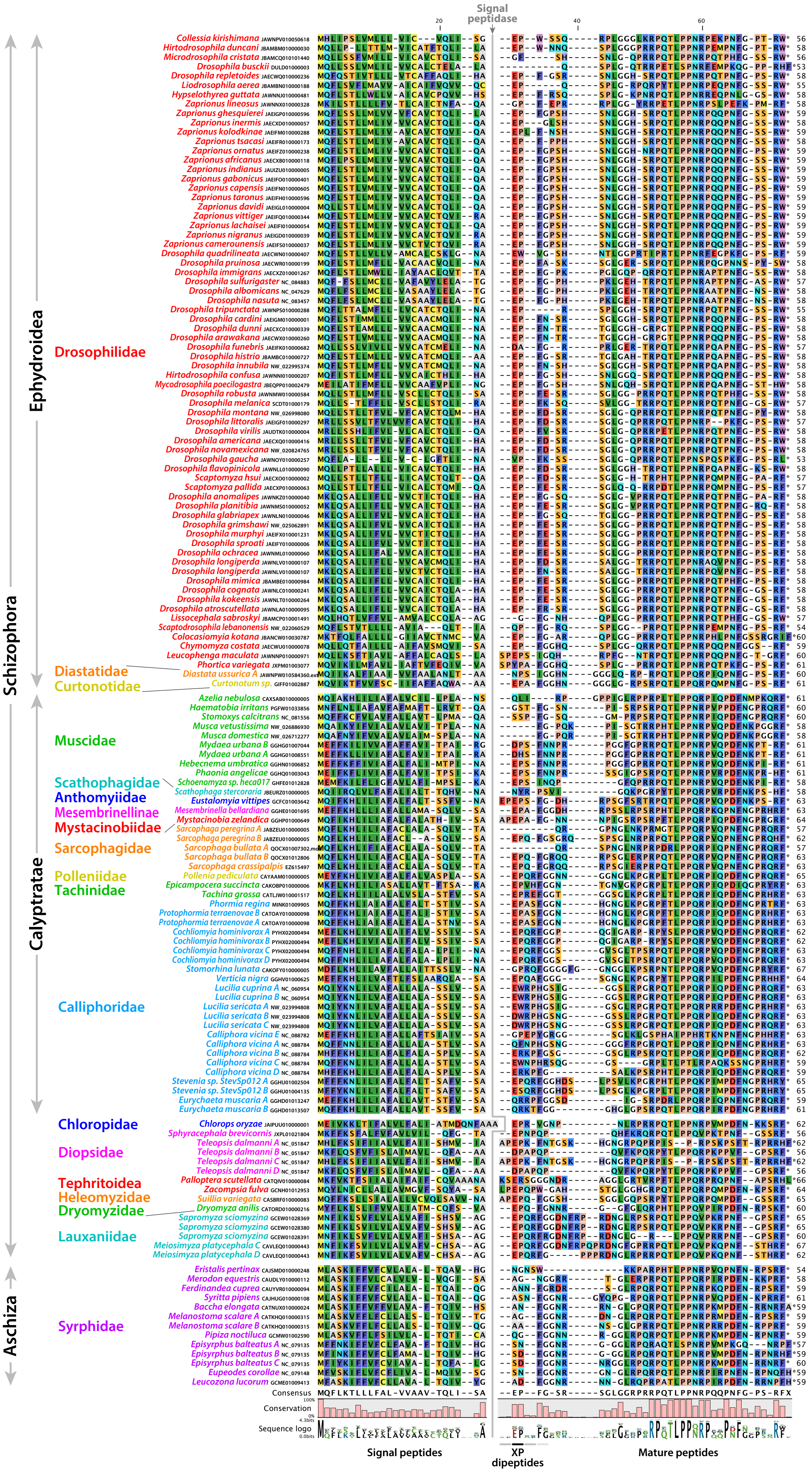

Sup. Fig. 3. Alignment of Mibin open reading frames across fly species

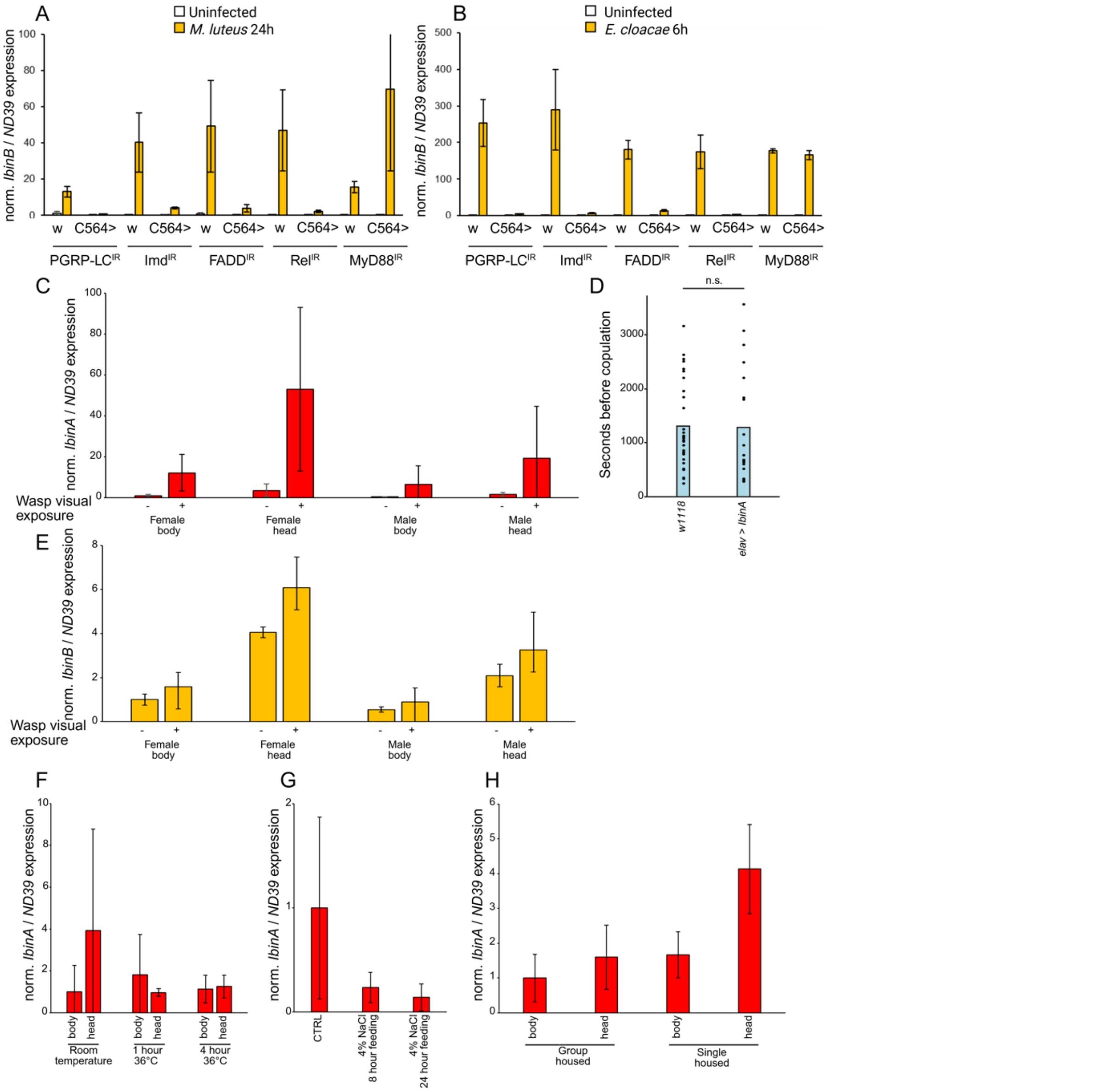

Sup. Fig. 4. *IbinB* expression is dependent on Imd components PGRP-LC, Imd, FADD and Rel, but not Toll pathway component MyD88, in the case of either *M. luteus* or *E. cloacae* infection. *IbinA* and *IbinB* do not show increased expression during physiological stress, but show higher expression during social isolation and exposure to parasitoid wasps. **A)** *IbinB* expression in female flies 24 hours post *M. luteus* infection with Imd and Toll pathway components knocked down. **B)** *IbinA* expression in female flies 24 hours post *M. luteus* infection with Imd and Toll pathway components knocked down. **C)** *IbinA* expression, determined by RT-qPCR, in heads and bodies of female and male flies visually exposed to *L. boulardi* wasps in neighboring vials. **D)** Time to copulation of pairs of male and female *D. melanogaster* comparing flies overexpressing *IbinA* under control of a neuron-specific driver to wild type. **E)** *IbinB*

expression, determined by RT-qPCR, in heads and bodies of female and male flies visually exposed to *L. boulardi* wasps in neighboring vials. **F)** *IbinA* expression in flies exposed to 36°C heat shock. **G)** *IbinA* expression following 4% NaCl feeding. **H)** *IbinA* expression in single housed (socially isolated) *D. melanogaster* vs. group housed flies. **C, E-H)** Canton-S flies were used in stress experiments.

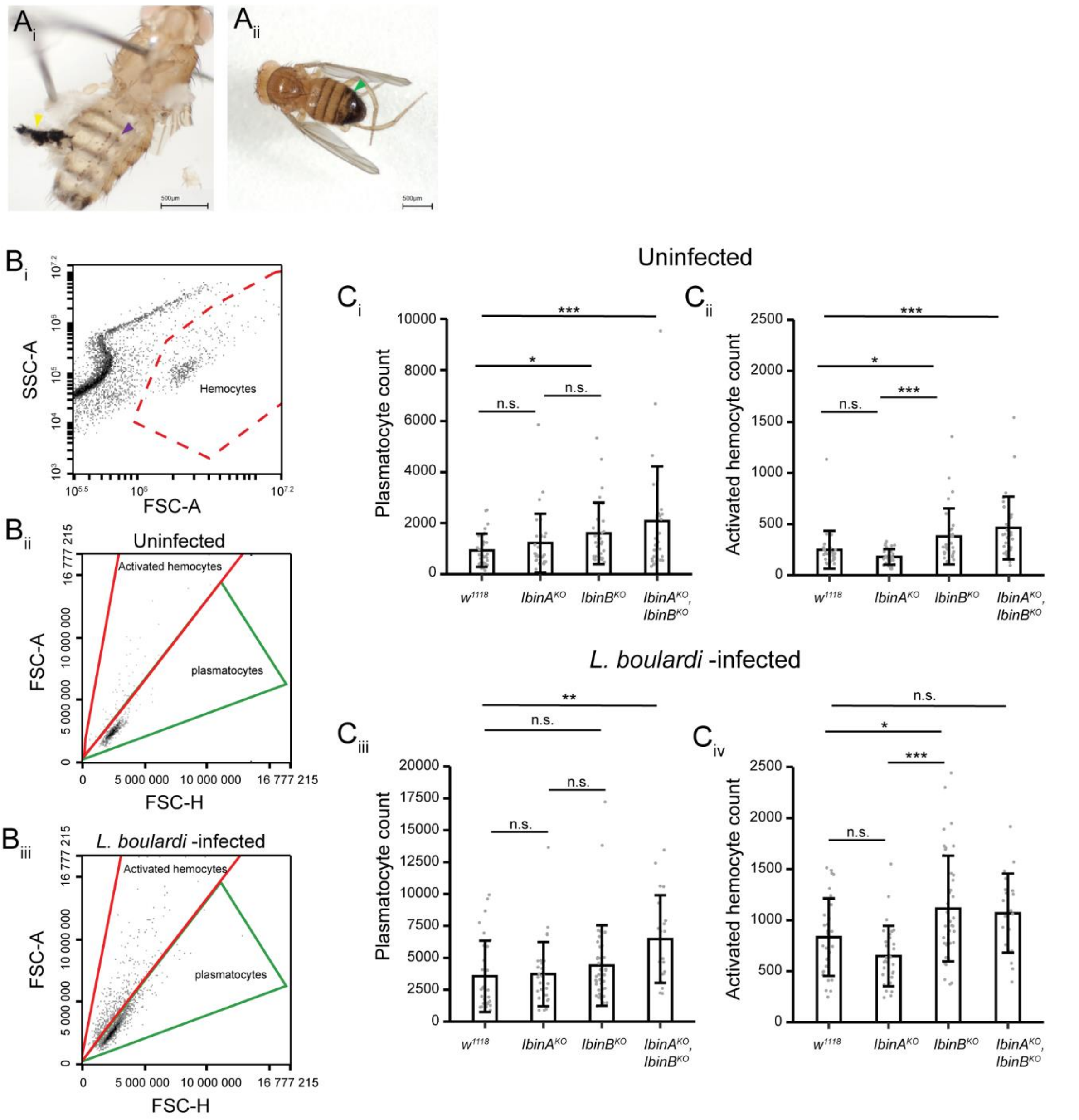

Sup. Fig. 5. Example images of Ibin mutant phenotype, flow cytometer gating strategy for hemocytes and hemocyte counts in Ibin mutant larvae. Ai-Aii) Besides the melanized spots,

lbin mutation also resulted in (Ai) brown coloration of cells on the dorsal side of the abdomen (purple arrowhead), putatively pericardial cells taking up melanized material and (Aii) a lack of melanization on the ventral striping pattern (green arrowhead). **Bi-Biii)** Gating strategy applied to hemocyte samples. **Bi)** Hemocytes form a population separated from cellular debris based on size and shape in the hemolymph on a forward scatter (FSC-A) vs. side scatter (SSC-A) area plot. Hemocytes in the gated area were used in subsequent analyses. **Bii-Biii)** hemocytes dissected from an uninfected (**Bii**) and *L. boulardi*-infected (**Biii**) larvae detected in the FSC-A vs. FSC-H plot. **Ci-Civ)** Quantification of hemocytes from uninfected (**Ci-Cii**) and wasp-infected (Ciii-Civ) larvae. n.s., not significant; * $p < 0.05$; ** $p< 0.01$; *** $p< 0.001$.

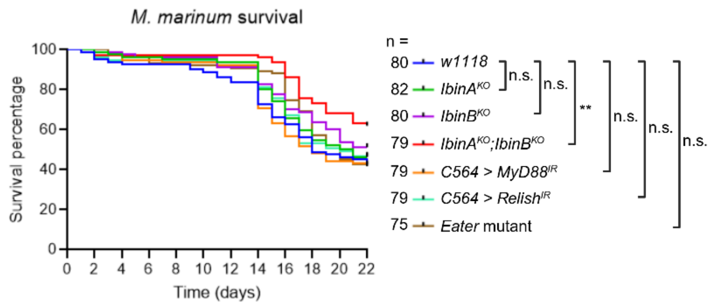

Sup. Fig. 6. *IbinA^{KO};IbinB^{KO}* flies have improved survival following *M. marinum* infection. Experiment includes fly lines deficient in the Toll pathway (*C564 > MyD88^{IR}*), in the Imd pathway (*C564 > Relish^{IR}*), and in phagocytosis (*Eater* mutant). Statistically significant differences are marked with asterisks: ** = p<0.01.

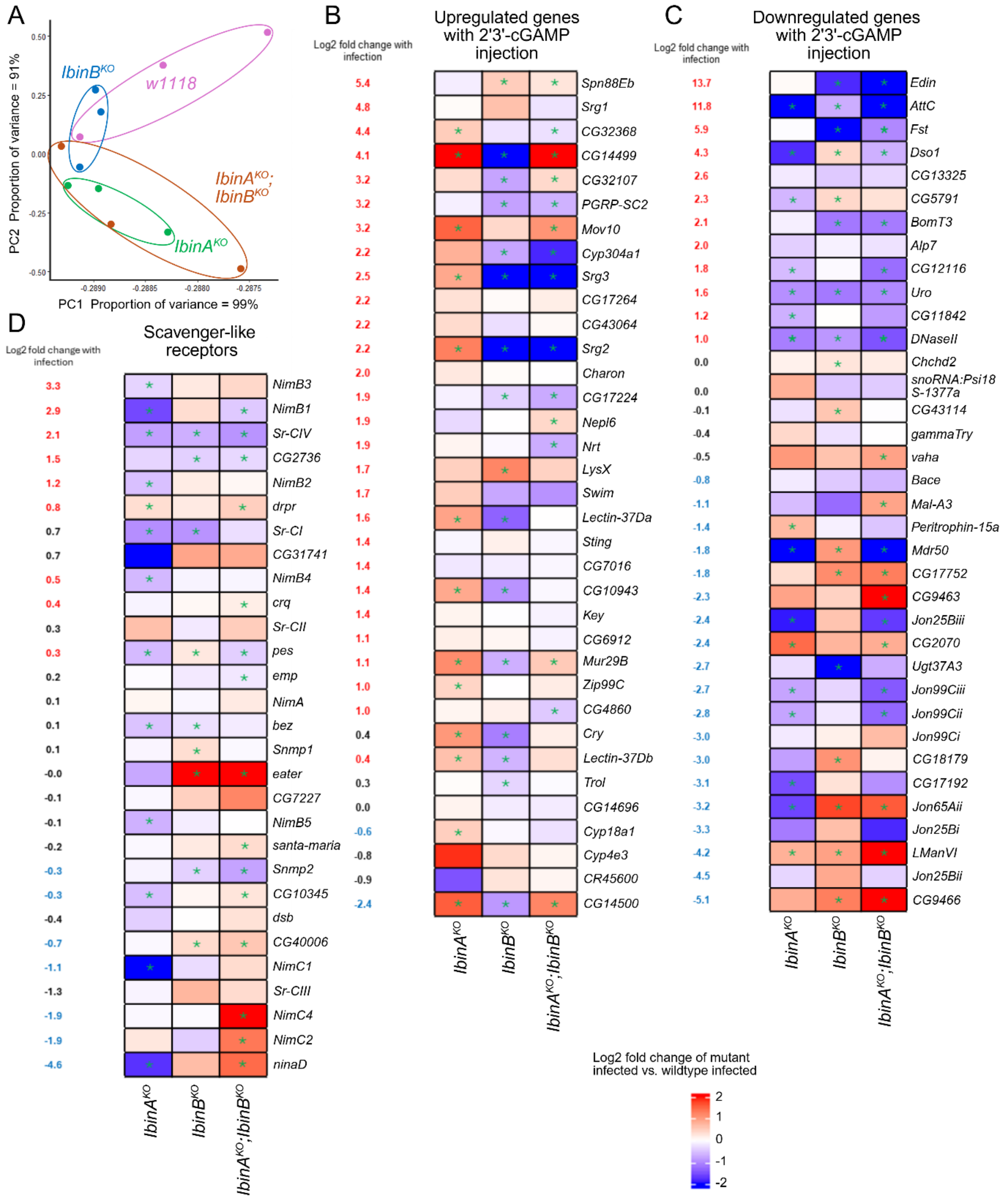

Sup. Fig. 7. Additional heatmaps of immune-relevant genes at 36hpi with *Listeria* in *IbinA*^{KO}, *IbinB*^{KO} and *IbinA*^{KO};*IbinB*^{KO} flies. A) PCA plot of RNA sequencing data of uninfected samples. **B)** Heatmap of genes **B)** upregulated or **C)** downregulated when flies are injected with 2'3'-cGAMP (Hedelin et al 2024). **D)** Heatmap of scavenger receptors. **B-D)** Colors signify Log2 fold change of infected mutant flies compared to infected wildtype flies. Green asterisk signifies adjusted p value < 0.05. Log2 fold change with infection numerical values compares infected wildtype to uninfected wildtype. Red and blue numerical values signify adjusted p value < 0.05.

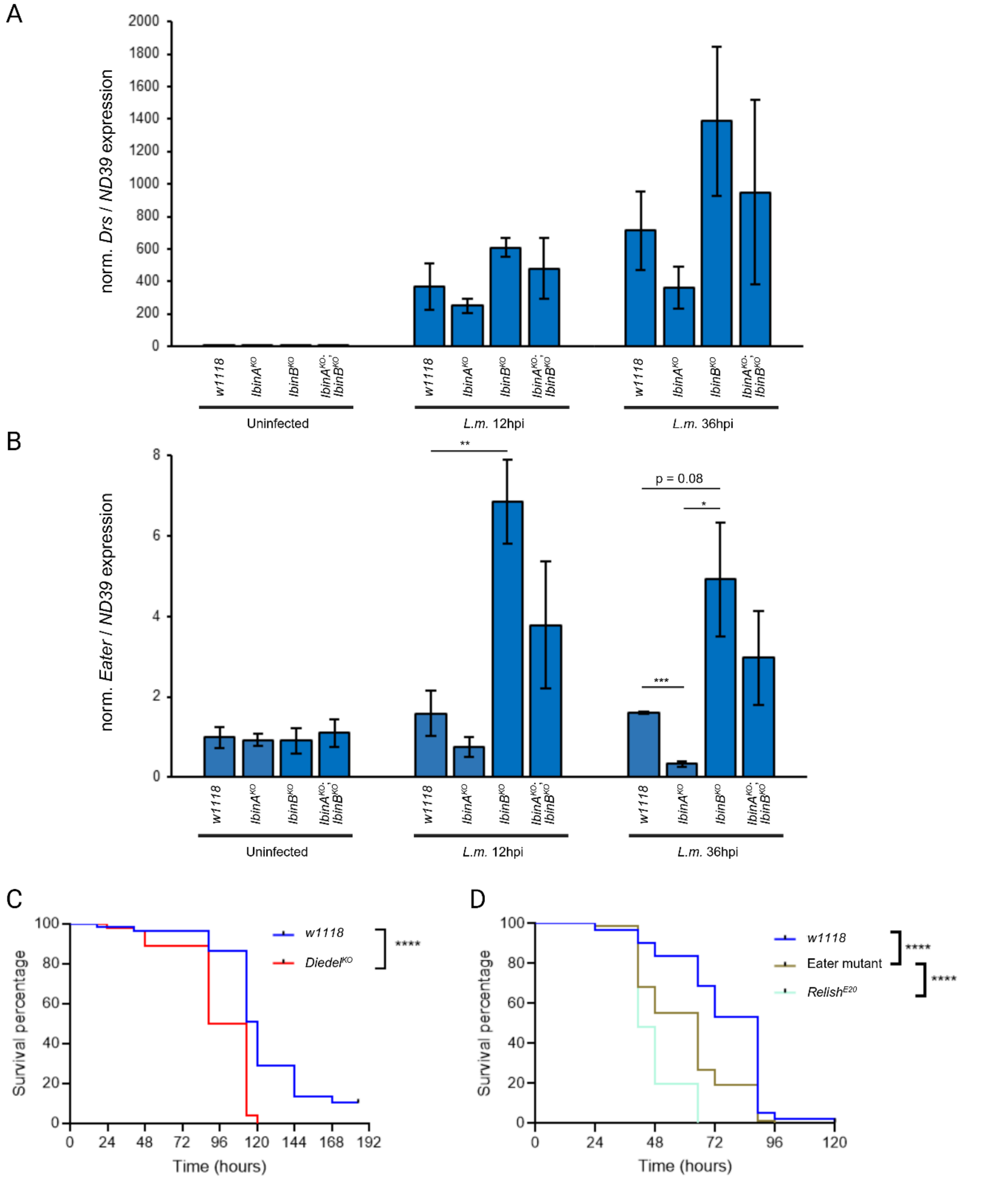

Sup. Fig. 8. qPCR verification of *Eater* and *Drs* expression at 36hpi with *Listeria*. Survival of *Eater* and *Diedel* mutant flies. A) Drs expression 12 and 36 hours post *L. monocytogenes* infection. **B)** *Eater* expression 12 and 36 hours post *L. monocytogenes* infection. **A-B)** For fold induction, *w1118* uninfected sample was set to 1. **C)** Survival of *Diedel* mutant flies following *L. monocytogenes* infection. **D)** Survival of *Eater* mutant flies following *L. monocytogenes* infection. *Relish*^{E20} positive control. **A-D)** Statistically significant differences are marked with asterisks: * = p<0.05; ** = p<0.01; *** = p<0.001, **** = p<0.0001.
